# Can frailty scores predict the incidence of cancer? Results from two large population-based studies

**DOI:** 10.1007/s11357-023-00783-9

**Published:** 2023-03-30

**Authors:** Jonathan K. L. Mak, Ralf Kuja-Halkola, Yunzhang Wang, Sara Hägg, Juulia Jylhävä

**Affiliations:** 1 Department of Medical Epidemiology and Biostatistics, Karolinska Institutet, Nobels Väg 12A, 171 77 Stockholm, Sweden; 2 Department of Clinical Sciences, Danderyd Hospital, Karolinska Institutet, Stockholm, Sweden; 3 Faculty of Social Sciences (Health Sciences) and Gerontology Research Center (GEREC), University of Tampere, Tampere, Finland

**Keywords:** Frailty, Cancer, Aging, Twins

## Abstract

While chronological age is the single biggest risk factor for cancer, it is less clear whether frailty, an age-related state of physiological decline, may also predict cancer incidence. We assessed the associations of frailty index (FI) and frailty phenotype (FP) scores with the incidence of any cancer and five common cancers (breast, prostate, lung, colorectal, melanoma) in 453,144 UK Biobank (UKB) and 36,888 Screening Across the Lifespan Twin study (SALT) participants, who aged 38–73 years and had no cancer diagnosis at baseline. During a median follow-up of 10.9 and 10.7 years, 53,049 (11.7%) and 4,362 (11.8%) incident cancers were documented in UKB and SALT, respectively. Using multivariable-adjusted Cox models, we found a higher risk of any cancer in frail vs. non-frail UKB participants, when defined by both FI (hazard ratio [HR] = 1.22; 95% confidence interval [CI] = 1.17–1.28) and FP (HR = 1.16; 95% CI = 1.11–1.21). The FI in SALT similarly predicted risk of any cancer (HR = 1.31; 95% CI = 1.15–1.49). Moreover, frailty was predictive of lung cancer in UKB, although this association was not observed in SALT. Adding frailty scores to models including age, sex, and traditional cancer risk factors resulted in little improvement in C-statistics for most cancers. In a within-twin-pair analysis in SALT, the association between FI and any cancer was attenuated within monozygotic but not dizygotic twins, indicating that it may partly be explained by genetic factors. Our findings suggest that frailty scores are associated with the incidence of any cancer and lung cancer, although their clinical utility for predicting cancers may be limited.

**Supplementary Information:**

The online version contains supplementary material available at 10.1007/s11357-023-00783-9.

## Introduction

Due to the global rise in life expectancy and that the incidence of most cancers increases with age [1], a better understanding on the risk factors of cancer is needed. While advancing age is the single most important risk factor for cancer overall and several cancer types [2], there is a large variability in the health status of older adults [3]. Independent of chronological age, other aging-related traits such as high blood pressure [4], sarcopenia [5], and impaired lung function [6], as well as biological age measures [7, 8], might be associated with the risk of cancer, too.

Frailty, a geriatric syndrome associated with multisystem dysfunction, is a manifestation of decline across several homeostatic systems [9]. It can also be considered as a measure of biological aging, such that it captures the heterogeneity in how individuals age [10]. Frailty correlates moderately with other markers of biological age, such as epigenetic clocks and telomere length, and explains a unique part of mortality risk not explained by other markers [11]. In addition to mortality, frailty is predictive of other adverse outcomes such as cardiovascular diseases [12], falls [13], hospitalizations [14], and disability [15]. Frailty is most commonly defined by the Rockwood frailty index (FI) [16] and the Fried frailty phenotype (FP) [17]. The FI is a multidimensional definition that operationalizes frailty as an accumulation of deficits across various health domains, such as diseases, symptoms, physical functioning, and mental well-being [16]. The FP views frailty as a physical syndrome with distinct physiological and functional manifestations characterized by weight loss, slowness, weakness, exhaustion, and low physical activity [17]. Despite the different definitions, both the FI and FP are valid predictors of adverse outcomes [18] and tap the same root causes of the syndrome [19]. The overall prevalence of frailty in individuals aged ≥ 50 is 24% using the FI and 12% using the FP [20].

Since cancer diagnosis and treatment are stressors that can deplete physiological reserves, frailty is of particular concern among cancer patients [21]. Studies have shown that 42% of older cancer patients are frail [22], and that frailty confers a high risk of chemotherapy intolerance, postoperative complications, disease progression, and mortality in cancer patients [21–23]. Having a cancer diagnosis may also lead to a higher risk of frailty [24], although the mechanisms are not understood. Meanwhile, there is a paucity of research on whether higher baseline frailty scores increase the risk of incident cancer. Existing literature mainly suggests no significant association between frailty and overall cancer incidence, yet most of these studies had relatively small sample sizes and did not analyze the risk of specific cancers [25–27]. As both frailty and cancer are closely linked to aging, we hypothesize that there could be a bidirectional relationship between frailty and cancer, such that a multisystem physiological dysregulation may also lead to an increased susceptibility to cancer risk. Improved understanding on the relationship between frailty and cancer may also help to inform clinical decisions and provide insights into the biological mechanisms underlying aging and cancer.

To this end, we aimed to assess whether higher baseline frailty scores predict the incidence of any cancer and the five most common cancers in Europe [28], namely breast, prostate, lung, colorectal, and melanoma skin cancer in two large population-based cohorts in the UK and Sweden. As a secondary aim, we used a co-twin control method to analyze whether the observed associations are explained by familial factors, i.e., genetics and shared environment.

## Methods

### Study population

Data were drawn from two prospective cohorts: the UK Biobank (UKB) study [29], and the Screening Across the Lifespan Twin (SALT) study in Sweden [30]. Both studies were approved by the local research ethics committees. All participants provided a written informed consent prior to data collection.

Between 2006 and 2010, postal invitations were sent to over 9 million adults registered with the UK’s National Health Service and lived close to one of the 22 assessment centers throughout England, Wales, and Scotland [31]. In total, > 500,000 participants aged 38–73 years from the general population were recruited to UKB (response rate 5.5%) [29, 31]. Participants completed a touch-screen questionnaire, had physical measurements taken, and provided biological samples during the baseline assessment. SALT is part of the population-based Swedish Twin Registry [30]. Between 1998 and 2002, all twins born in 1958 or before were invited to participate in the survey with an aim to screen for common diseases [32]. In total, 44,919 twin individuals aged 41–103 years were recruited (response rate was 65% for those born in 1886–1925, and 74% for those born in 1926–1958) [30, 32]. SALT participants completed a computer-assisted telephone interview at baseline with questions on diseases, symptoms, medication use and lifestyle.

In this analysis, we excluded UKB and SALT participants who had a history of cancer diagnosis (except non-melanoma skin cancer) before baseline and those with missing data on frailty. For feasible comparison, we further excluded SALT participants aged > 73 years at baseline to match with the age range of UKB participants. In total, we included 453,144 participants from UKB and 36,888 participants from SALT in the analytical samples (Supplementary Fig. 1).

### Frailty assessment

Frailty was measured using the FI and FP in UKB, and only the FI in SALT. Following the deficit accumulation model [16], we have previously constructed and validated FIs for both cohorts [33, 34]. Briefly, 49 and 44 self-reported deficit items, including signs, symptoms, and diseases in various physiological and mental domains, were selected for construction of the FIs in UKB and SALT, respectively (Supplementary Table 1). Participants who had missing data on > 20% of the frailty items were excluded. We calculated the FI as the sum of deficits divided by the number of non-missing items (e.g., an individual with 7 deficits out of 44 items would receive an FI of 7/44 = 0.16). Following the cut-offs that have been used in our previous work [34, 35], we considered four FI categories: relatively fit (≤ 0.03), less fit (> 0.03–0.1), least fit (> 0.1–0.21), and frail (> 0.21). The FI was used as both continuous (per 10% increase) and categorical variables in the analysis.

A modified FP was previously created for UKB participants using the five frailty criteria [17]: weight loss, exhaustion, slowness, low physical activity, and weakness [36]. The first four criteria were assessed by self-reported questionnaires, and weakness was determined by the grip strength measured using a Jamar J00105 hydraulic hand dynamometer (Supplementary Table 2). Participants were categorized into non-frail (met none of the five criteria), pre-frail (1–2), and frail (≥ 3) [17]. We excluded those who had missing data on any of the five criteria.

### Cancer ascertainment

Incident cancers in UKB were identified through a linkage to the UK national cancer registries; complete follow-up was available from the baseline through February 29, 2020. Incident cancers in SALT were identified through a linkage to the Swedish National Patient Register; we used a comparable follow-up period as in UKB and followed SALT participants up to December 31, 2011. We defined cancers according to the International Classification of Diseases, 10^th^ revision codes: breast cancer (C50), prostate cancer (C61), lung cancer (including trachea, C33-34), colorectal cancer (C18-20), melanoma of skin (C43); and any cancer diagnosis, excluding non-melanoma skin cancer (C00-97, except C44). The analysis on breast and prostate cancer was performed only in women and men, respectively.

### Covariates

We considered age, birth year, sex, baseline assessment center, ethnic background, body mass index (BMI), smoking, alcohol consumption, education, and deprivation index as the common confounders for all cancer sites in UKB. BMI was derived from the weight and height measured by trained nurses at baseline. These variables, except baseline assessment center, ethnic background, and deprivation index, were also available and adjusted for in SALT. We also considered the following covariates for site-specific cancers in UKB: models for breast, prostate, lung, and colorectal cancer were additionally adjusted for family history of the corresponding cancer, while the models for melanoma were additionally adjusted for physical activity, time spent outdoors during summer, use of ultraviolet protection, childhood sunburns, solarium/sunlamp use, ease of skin tanning, skin color, and hair color (these variables were not available in SALT). Family history of melanoma was not available in UKB. Details on the categorization and descriptive statistics of the covariates are presented in Supplementary Tables 3–4.

### Statistical analysis

Analyses were performed separately in UKB and SALT. Participants were followed from the date of baseline assessment to the date of cancer diagnosis, death, or end of follow-up, whichever came first.

***Survival analysis.*** Kaplan–Meier curves were first plotted to evaluate the probabilities of cancer incidence across frailty categories. To account for the competing risk of death, we also calculated cumulative incidences of the cancers using the Aalen-Johansen estimator [37].

We calculated hazard ratios (HRs) and 95% confidence intervals (CIs) using Cox proportional-hazard models, with attained age as the underlying timescale. All models were adjusted for sex, birth year, and the other covariates described above, as relevant for each cancer. Indicator variables for the missing data of covariates were created and included in the models when necessary. We also assessed whether frailty was associated with the two most common subtypes of lung cancer, adenocarcinoma and squamous cell carcinoma, in UKB. Cluster-robust standard errors were used in SALT to account for the relatedness of individuals in twin pairs. Discrimination ability of the Cox models was assessed using the Harrell’s C-statistics [38].

For the cancers that had statistically significant associations with the baseline frailty scores in both cohorts, we additionally stratified the analysis by baseline age (< 60 vs. ≥ 60 years), sex, BMI (< 25 vs. ≥ 25), and smoking status (non-smokers vs. ever-smokers). We also performed several sensitivity analyses. First, we tested for non-proportional hazards over time by fitting flexible parametric survival models, where the baseline hazard function was modelled using a 5 degrees-of-freedom natural cubic spline, and the time-dependent effect of frailty using a 3 degrees-of-freedom spline. Second, since the FIs included self-reported cancer items (two items in UKB and one in SALT), we removed these items from the FIs to assess whether they had any effect on the results. Similarly, as we observed an association between the FI and lung cancer in UKB, we removed seven lung cancer-related items from the UKB FI (i.e., wheezing, pneumonia, chronic lung disease, asthma, chest pain, any cancer diagnosis, and multiple cancers diagnosed) to examine whether the association was influenced by these items. Finally, instead of using the missing-indicator approach, we performed a complete-case analysis by excluding individuals with missing data on any covariates from the Cox models.

***Within-twin-pair analysis.*** In SALT, we fitted stratified Cox models within dizygotic (DZ) and monozygotic (MZ) twin pairs to examine whether the significant associations observed in the population-level analysis were due to familial factors [39]. The co-twin control design rests on the assumption that MZ twins are genetically identical and DZ twins share ~ 50% of their segregating genes. Both MZ and DZ twins share the same family environment (early rearing environment and everything that makes twins in a pair similar to each other). If the association is not affected by familial influences (i.e., in line with a causal hypothesis), the effect size should remain similar in the within-twin-pair analysis compared to the population-level analysis [39]. In contrast, if the association between frailty and cancer is explained by genetic factors, we would expect an attenuation of the association in DZ twins, and an even greater attenuation in MZ twins. If the association is explained by shared environmental factors, a similar attenuation is in both DZ and MZ twins.

To account for multiple comparisons (two frailty measures and five cancer sites), we applied the Bonferroni adjustment and considered *p* < 0.005 (0.05/10) as statistically significant. All analyses were performed in Stata 16 and R 4.0.5.

## Results

### Sample characteristics

For the included 453,144 UKB participants and 36,888 SALT participants, the median follow-up periods for any cancer were 10.9 years (interquartile range [IQR] 10.0–11.6) and 10.7 years (IQR 9.7–11.9), respectively. A total of 53,049 (11.7%) incident cancers were documented in UKB and 4,362 (11.8%) in SALT. The cumulative incidence of the site-specific cancers was similar in both cohorts (Table 1). The mortality rate in SALT was higher than that in UKB (9.7% vs. 5.0%), possibly because SALT participants were mostly from an older birth cohort than UKB participants. The proportion of frail individuals defined by FI was 11.9% in UKB and 14.4% in SALT, and by FP was 3.6% in UKB (Table 1). There was a weak-to-moderate correlation between FI and FP in UKB (Spearman’s correlation = 0.35) (Supplementary Fig. 2).

**Table 1:** Characteristics of study participants. Data are numbers (%) unless otherwise indicated

Variable	UK Biobank (*n* = 453,144)	SALT (*n* = 36,888)
Age at baseline, mean ± SD	56.3 ± 8.1	56.1 ± 8.1
Year of birth		
< 1930	-	2,994 (8.1)
1930–1939	15,272 (3.4)	8,654 (23.5)
1940–1949	192,258 (42.4)	13,956 (37.8)
1950–1959	147,680 (32.6)	11,284 (30.6)
≥ 1960	97,934 (21.6)	-
Women	241,075 (53.2)	19,209 (52.1)
Frailty index, median (IQR)^a^	0.107 (0.066, 0.163)	0.102 (0.057, 0.165)
Relatively fit (≤ 0.03)	28,775 (6.4)	3,805 (10.3)
Less fit (> 0.03 to 0.1)	173,252 (38.2)	14,090 (38.2)
Least fit (> 0.1 to 0.21)	197,338 (43.5)	13,665 (37.0)
Frail (> 0.21)	53,779 (11.9)	5,328 (14.4)
Frailty phenotype, mean (SD)^b^	0.59 ± 0.84	-
Non-frail (0)	262,830 (58.0)	-
Pre-frail (1 to 2)	173,834 (38.4)	-
Frail (3 to 5)	16,480 (3.6)	-
Body mass index		
Underweight (< 18.5)	2,282 (0.5)	419 (1.1)
Normal weight (18.5 to < 25)	147,310 (32.5)	19,022 (51.6)
Overweight (25 to < 30)	193,461 (42.7)	13,769 (37.3)
Obese (≥ 30)	110,091 (24.3)	3,048 (8.3)
Missing	-	630 (1.7)
Smoking status		
Never	249,213 (55.0)	14,512 (39.3)
Previous	155,088 (34.2)	14,206 (38.5)
Current	47,294 (10.4)	8,033 (21.8)
Missing	1,549 (0.3)	137 (0.4)
Alcohol intake frequency		
Less than weekly	136,987 (30.2)	10,383 (28.1)
Weekly	315,820 (69.7)	24,321 (65.9)
Missing	337 (0.1)	2,184 (5.9)
Education level		
High	148,885 (32.9)	9,617 (26.1)
Intermediate	224,777 (49.6)	17,977 (48.7)
Low	75,047 (16.6)	9,117 (24.7)
Missing	4,435 (1.0)	177 (0.5)
Died during follow-up	22,879 (5.0)	3,580 (9.7)
Any incident cancer^c^	53,049 (11.7)	4,362 (11.8)
Incident breast cancer in women^d^	8,495 (3.5)	699 (3.6)
Incident prostate cancer in men^e^	10,588 (5.0)	1,059 (6.0)
Incident lung cancer	3,685 (0.8)	338 (0.9)
Incident colorectal cancer	5,424 (1.2)	438 (1.2)
Incident melanoma	2,731 (0.6)	195 (0.5)

*IQR* interquartile range, *SALT* Screening Across the Lifespan Twin Study, *SD* standard deviation

^a^A 49-item frailty index was used in UK Biobank, and a 44-item frailty index was used in SALT

^b^The frailty phenotype was not available in SALT

^c^Non-melanoma skin cancer was not included

^d^Denominators for breast cancer percentage calculations are of women only

^e^Denominators for prostate cancer percentage calculations are of men only

### Frailty and risk of cancer

**Fig. 1:**
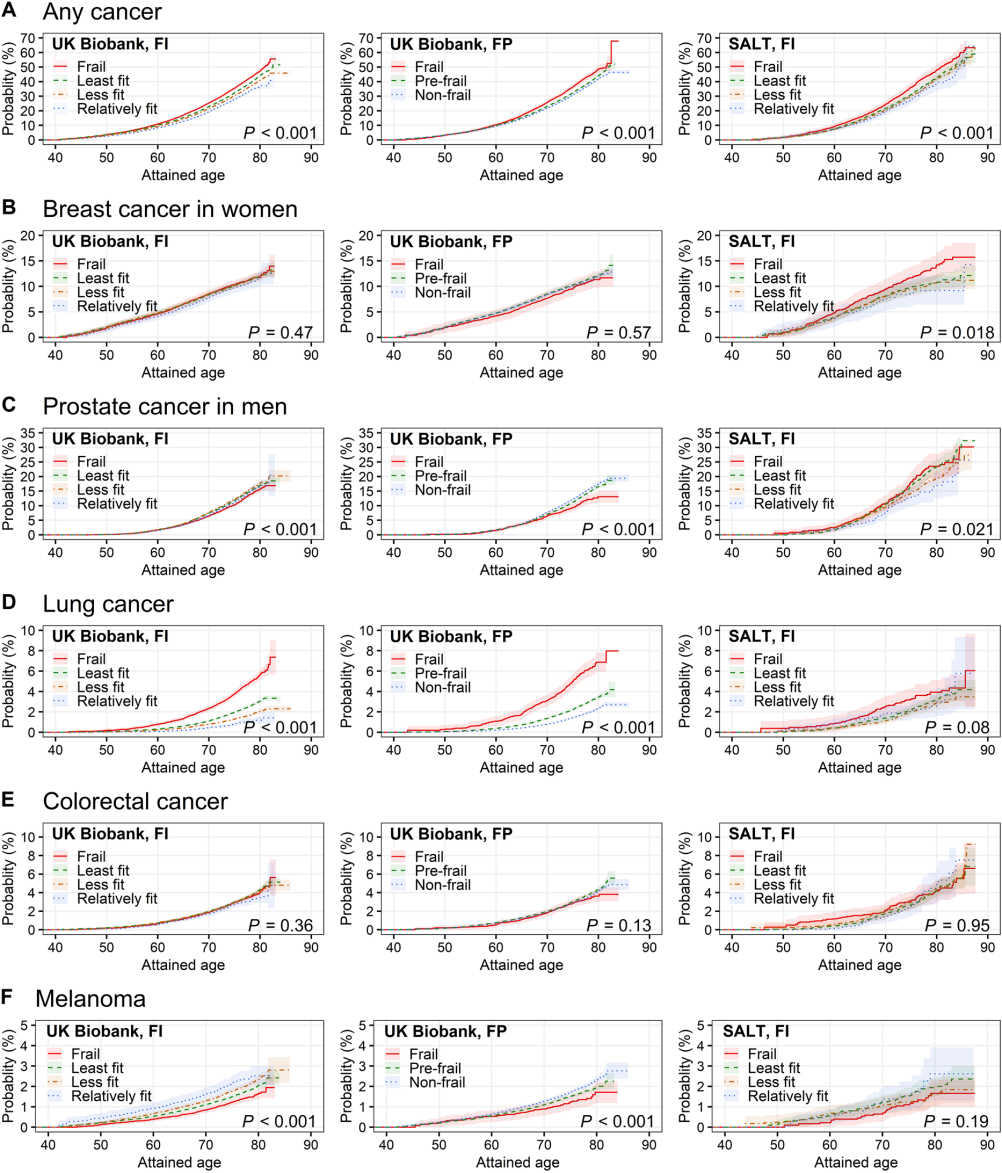
Probability of cancer incidence over attained age, stratified by frailty status in UK Biobank and SALT. **A** Incidence of any cancer, except non-melanoma skin cancer; **B** incidence of breast cancer in women; **C** incidence of prostate cancer in men; **D** incidence of lung cancer; **E** incidence of colorectal cancer; **F** incidence of melanoma. Frailty was assessed using a 49-item FI and a modified FP in UK Biobank, and by a 44-item FI in SALT. Probability of cancer incidence was based on the Kaplan–Meier estimates. *P*-values were based on log-rank tests. Abbreviations: FI, frailty index; FP, frailty phenotype; SALT, Screening Across the Lifespan Twin Study

Kaplan–Meier curves showed that higher baseline frailty scores were associated with an increased probability of any cancer in both cohorts, and a higher probability of lung cancer and a lower probability of prostate cancer and melanoma in UKB (log-rank *p* < 0.005) (Fig. 1). Results were similar when accounted for the competing risk of death, except for that the cumulative incidence of any cancer appeared to be lower in frail individuals defined by the UKB FP, possibly because the most frail individuals may have already died before getting cancers during the follow-up (Supplementary Fig. 3).

In multivariable-adjusted Cox models, we observed significant associations for baseline FI (HR for frail vs. relatively fit = 1.22; 95% CI = 1.17–1.28) and FP scores (HR for frail vs. non-frail = 1.16; 95% CI = 1.11–1.21) with any cancer in UKB (Table 2). Similarly, in SALT, frail vs. relatively fit participants as defined by the FI also had an increased risk of any cancer (HR = 1.31; 95% CI = 1.15–1.49) (Table 3). For specific cancer sites in UKB, higher FI and FP scores were associated with increased risks of all lung cancer (Table 2), as well as its subtypes, adenocarcinoma and squamous cell carcinoma, although the association between FP and adenocarcinoma was not statistically significant in the fully-adjusted model (Supplementary Table 5). Higher FI was also associated with a reduced risk of melanoma in UKB, although no significant association was observed between FP and melanoma (Table 2). The FI was not statistically significantly associated with the specific cancers in SALT after accounting for multiple comparisons (Table 3).

**Table 2:** Associations between baseline frailty scores by the frailty index and frailty phenotype and cancer incidence in UK Biobank (*n* = 453,144). Data are hazard ratios (95% confidence intervals) unless otherwise indicated

Cancer site	Frailty index					Frailty phenotype		
	Relatively fit	Less fit	Least fit	Frail	Per 10% increase	Non-frail	Pre-frail	Frail
Any cancer								
Incidence per 100,000 person-years	879.2	1043.8	1176.4	1371.0	-	1085.1	1170.4	1401.7
Age- and sex-adjusted model^a^	1 (ref.)	1.11 (1.06, 1.15)*	1.18 (1.13, 1.23)*	1.31 (1.26, 1.37)*	1.10 (1.09, 1.11)*	1 (ref.)	1.07 (1.05, 1.09)*	1.23 (1.18, 1.29)*
Multivariable model^b^	1 (ref.)	1.09 (1.05, 1.13)*	1.13 (1.09, 1.18)*	1.22 (1.17, 1.28)*	1.07 (1.07, 1.08)*	1 (ref.)	1.05 (1.03, 1.07)*	1.16 (1.11, 1.21)*
Breast cancer in women								
Incidence per 100,000 person-years	302.7	324.6	323.7	345.7	-	322.6	330.6	319.4
Age-adjusted model^a^	1 (ref.)	1.04 (0.94, 1.15)	1.02 (0.92, 1.13)	1.07 (0.95, 1.19)	1.01 (0.98, 1.04)	1 (ref.)	1.02 (0.97, 1.06)	0.97 (0.87, 1.08)
Multivariable model^b^	1 (ref.)	1.03 (0.93, 1.14)	1.01 (0.91, 1.11)	1.05 (0.94, 1.18)	1.01 (0.98, 1.04)	1 (ref.)	1.01 (0.97, 1.06)	0.97 (0.87, 1.09)
Breast cancer-specific model^c^	1 (ref.)	1.03 (0.93, 1.14)	1.00 (0.91, 1.11)	1.05 (0.94, 1.18)	1.01 (0.98, 1.04)	1 (ref.)	1.01 (0.97, 1.06)	0.97 (0.87, 1.09)
Prostate cancer in men								
Incidence per 100,000 person-years	380.9	467.5	494.2	471.6	-	485.1	456.2	415.0
Age-adjusted model^a^	1 (ref.)	1.09 (1.01, 1.19)	1.05 (0.96, 1.14)	0.94 (0.85, 1.03)	0.95 (0.93, 0.98)*	1 (ref.)	0.92 (0.89, 0.96)*	0.76 (0.67, 0.86)*
Multivariable model^b^	1 (ref.)	1.11 (1.03, 1.21)	1.10 (1.01, 1.20)	1.06 (0.96, 1.17)	1.00 (0.97, 1.03)	1 (ref.)	0.96 (0.92, 1.00)	0.86 (0.76, 0.98)
Prostate cancer-specific model^d^	1 (ref.)	1.11 (1.02, 1.21)	1.10 (1.01, 1.20)	1.05 (0.95, 1.16)	1.00 (0.97, 1.03)	1 (ref.)	0.96 (0.92, 1.00)	0.86 (0.76, 0.98)
Lung cancer								
Incidence per 100,000 person-years	26.0	49.2	81.2	163.9	-	56.8	91.2	201.3
Age- and sex-adjusted model^a^	1 (ref.)	1.69 (1.35, 2.12)*	2.55 (2.04, 3.18)*	4.84 (3.86, 6.06)*	1.68 (1.62, 1.74)*	1 (ref.)	1.58 (1.47, 1.69)*	3.30 (2.94, 3.71)*
Multivariable model^b^	1 (ref.)	1.47 (1.17, 1.84)*	1.86 (1.49, 2.32)*	2.71 (2.15, 3.41)*	1.36 (1.31, 1.42)*	1 (ref.)	1.31 (1.23, 1.41)*	1.94 (1.72, 2.20)*
Lung cancer-specific model^e^	1 (ref.)	1.47 (1.17, 1.84)*	1.85 (1.48, 2.31)*	2.68 (2.13, 3.37)*	1.36 (1.30, 1.41)*	1 (ref.)	1.31 (1.22, 1.40)*	1.92 (1.70, 2.17)*
Colorectal cancer								
Incidence per 100,000 person-years	86.7	105.1	116.2	121.6	-	107.1	115.8	114.2
Age- and sex-adjusted model^a^	1 (ref.)	1.13 (0.99, 1.28)	1.16 (1.03, 1.32)	1.16 (1.01, 1.34)	1.02 (0.98, 1.05)	1 (ref.)	1.08 (1.02, 1.14)	1.03 (0.89, 1.19)
Multivariable model^b^	1 (ref.)	1.10 (0.97, 1.25)	1.11 (0.98, 1.26)	1.08 (0.93, 1.25)	0.99 (0.95, 1.03)	1 (ref.)	1.06 (1.01, 1.13)	1.01 (0.87, 1.17)
Colorectal cancer-specific model^f^	1 (ref.)	1.10 (0.97, 1.25)	1.11 (0.98, 1.26)	1.07 (0.93, 1.24)	0.98 (0.95, 1.02)	1 (ref.)	1.06 (1.01, 1.13)	1.00 (0.86, 1.16)
Melanoma								
Incidence per 100,000 person-years	61.6	59.3	53.7	47.4	-	58.6	52.3	41.5
Age- and sex-adjusted model^a^	1 (ref.)	0.91 (0.78, 1.06)	0.79 (0.68, 0.92)*	0.67 (0.56, 0.81)*	0.85 (0.81, 0.90)*	1 (ref.)	0.89 (0.82, 0.96)*	0.68 (0.54, 0.87)*
Multivariable model^b^	1 (ref.)	0.92 (0.79, 1.08)	0.83 (0.71, 0.97)	0.77 (0.64, 0.93)	0.90 (0.85, 0.96)*	1 (ref.)	0.96 (0.88, 1.04)	0.88 (0.69, 1.12)
Melanoma-specific model^c^	1 (ref.)	0.91 (0.78, 1.06)	0.81 (0.69, 0.94)	0.75 (0.62, 0.91)*	0.89 (0.84, 0.95)*	1 (ref.)	0.97 (0.90, 1.05)	0.94 (0.73, 1.20)

^a^Age- and sex-adjusted model: adjusted for age (time scale), birth year, and sex (except for breast cancer and prostate cancer)

^b^Multivariable model: age- and sex-adjusted model + baseline assessment center, ethnic background, body mass index, smoking status, alcohol consumption, education level, deprivation index quintiles

^c^Breast cancer-specific model: multivariable model + family history of breast cancer

^d^Prostate cancer-specific model: multivariable model + family history of prostate cancer

^e^Lung cancer-specific model: multivariable model + family history of lung cancer

^f^Colorectal cancer-specific model: multivariable model + family history of colorectal cancer

^g^Melanoma cancer-specific model: multivariable model + physical activity level, time spent outdoors during summer, use of sun/UV protection, sunburn during childhood, solarium/sunlamp use, ease of skin tanning, skin color, hair color

^*^ Significant after Bonferroni adjustment at *p* < .005 (i.e., .05/10, considering 2 frailty measures × 5 cancers)

**Table 3:** Associations between frailty index and cancer incidence in SALT (*n* = 36,888). Data are hazard ratios (95% confidence intervals) unless otherwise indicated

Cancer site	Frailty index				
	Relatively fit	Less fit	Least fit	Frail	Per 10% increase
Any cancer					
Incidence per 100,000 person-years	903.0	1054.3	1202.3	1460.8	-
Age- and sex-adjusted model^a^	1 (ref.)	1.08 (0.97, 1.21)	1.16 (1.03, 1.29)	1.34 (1.18, 1.52)*	1.09 (1.05, 1.12)*
Multivariable model^b^	1 (ref.)	1.08 (0.96, 1.21)	1.14 (1.01, 1.28)	1.31 (1.15, 1.49)*	1.08 (1.04, 1.12)*
Breast cancer in women					
Incidence per 100,000 person-years	273.7	323.3	328.5	429.5	-
Age- and sex-adjusted model^a^	1 (ref.)	1.15 (0.85, 1.56)	1.16 (0.85, 1.57)	1.49 (1.08, 2.05)	1.11 (1.03, 1.20)
Multivariable model^b^	1 (ref.)	1.14 (0.84, 1.55)	1.15 (0.84, 1.56)	1.49 (1.08, 2.07)	1.11 (1.03, 1.20)
Prostate cancer in men					
Incidence per 100,000 person-years	387.2	520.1	667.3	740.2	-
Age- and sex-adjusted model^a^	1 (ref.)	1.20 (0.95, 1.52)	1.38 (1.09, 1.75)	1.38 (1.05, 1.82)	1.07 (1.00, 1.15)
Multivariable model^b^	1 (ref.)	1.21 (0.96, 1.53)	1.42 (1.13, 1.80)*	1.47 (1.12, 1.94)	1.10 (1.02, 1.19)
Lung cancer					
Incidence per 100,000 person-years	80.9	67.8	92.2	119.8	-
Age- and sex-adjusted model^a^	1 (ref.)	0.77 (0.52, 1.14)	0.96 (0.65, 1.40)	1.14 (0.75, 1.74)	1.14 (1.02, 1.29)
Multivariable model^b^	1 (ref.)	0.76 (0.51, 1.11)	0.87 (0.60, 1.27)	0.98 (0.64, 1.50)	1.08 (0.95, 1.22)
Colorectal cancer					
Incidence per 100,000 person-years	93.3	105.7	117.7	121.9	-
Age- and sex-adjusted model^a^	1 (ref.)	1.01 (0.71, 1.44)	1.01 (0.71, 1.43)	0.95 (0.64, 1.43)	0.93 (0.83, 1.04)
Multivariable model^b^	1 (ref.)	1.01 (0.71, 1.44)	0.99 (0.69, 1.41)	0.92 (0.61, 1.39)	0.91 (0.82, 1.03)
Melanoma					
Incidence per 100,000 person-years	58.9	40.9	56.8	46.5	-
Age- and sex-adjusted model^a^	1 (ref.)	0.67 (0.42, 1.07)	0.90 (0.57, 1.43)	0.73 (0.41, 1.27)	0.99 (0.84, 1.17)
Multivariable model^b^	1 (ref.)	0.67 (0.42, 1.08)	0.93 (0.59, 1.47)	0.77 (0.44, 1.36)	1.01 (0.85, 1.20)

*SALT* Screening Across the Lifespan Twin Study

^a^Age- and sex-adjusted model: adjusted for age (time scale), birth year, and sex (except for breast cancer and prostate cancer)

^b^Multivariable model: age- and sex-adjusted model + body mass index, smoking status, alcohol consumption, and education level

^*^Significant after Bonferroni adjustment at *p* < .005 (i.e., .05/10, considering 2 frailty measures × 5 cancers)

We examined the discrimination ability of frailty scores for cancers using the Harrell’s C-statistics (Supplementary Table 6). Compared to the models including age, sex, and traditional cancer risk factors as explanatory variables, adding frailty scores did not improve discrimination for any cancer and most cancer types. There was only slight improvement in the C-statistic (0.784 vs. 0.779) when adding FI to the model including common risk factors (e.g., age, sex, and smoking) in predicting lung cancer in UKB (Supplementary Table 6).

In subgroup analysis, the associations between baseline frailty scores and incidence of any cancer were stronger in men vs. women (*P*_interaction_ < 0.001 for FI and *P*_interaction_ = 0.040 for FP) and in ever-smokers vs. non-smokers (*P*_interaction_ < 0.001 for both FI and FP) in UKB (Supplementary Table 7). No significant interactions were observed across the subgroups in SALT (Supplementary Table 8).

When allowing for time-varying hazards, we observed relatively stable associations between both the FI and FP and any cancer over the follow-up period in UKB (Supplementary Fig. 4). However, in SALT, the association between FI and any cancer was the strongest at approximately one year after baseline and was attenuated after four years since follow-up (Supplementary Fig. 4). The association between FI and any cancer remained essentially unchanged after removing self-reported cancer items from the FI in both samples (Supplementary Table 9). Moreover, when removing lung cancer-related items from the FI in UKB, the FI-lung cancer association remained robust and statistically significant (Supplementary Table 10). Finally, we observed largely consistent results in the complete-case analysis (Supplementary Table 11).

### Within-twin-pair analysis

**Fig. 2:**
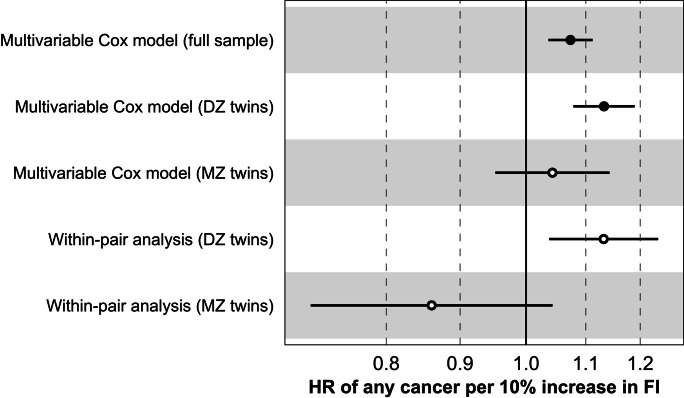
Association between the frailty index and risk of any cancer from the multivariable Cox models and with-pair analyses in the full SALT cohort (*n* = 36,888), complete DZ twin pairs (*n* = 9,920 pairs) and complete MZ twin pairs (*n* = 3,610 pairs). Error bars represent the 95% confidence intervals. Closed symbols represent *p* < 0.005. Abbreviations: DZ, dizygotic; FI, frailty index; MZ, monozygotic; SALT, Screening Across the Lifespan Twin Study

To test whether the association between FI and risk of any cancer in SALT was due to shared familial confounding, we performed a within-twin-pair analysis in DZ and MZ twins. We first obtained population-level estimates in multivariable Cox models for DZ (HR per 10% increase in FI = 1.13; 95% CI = 1.08–1.19) and MZ twins (HR per 10% increase in FI = 1.04; 95% CI = 0.95–1.14) and found that they were comparable to the population-level estimate in the full SALT sample (Fig. 2). Compared to population-level estimates, the association between FI and risk of any cancer was similar in the within-pair analysis in DZ twin pairs (HR per 10% increase in FI = 1.13; 95% CI = 1.04–1.24), but was attenuated in MZ pairs (HR per 10% increase in FI = 0.86; 95% CI = 0.71–1.04) (Fig. 2).

## Discussion

Using data from two large population-based cohorts, we showed that higher baseline frailty scores, measured using the FI and FP, were associated with an increased risk of any cancer after adjusting for common cancer risks such as age, sex, BMI, smoking, alcohol consumption and education. We also observed an elevated risk of lung cancer and its two main subtypes, adenocarcinoma and squamous cell carcinoma, with higher frailty scores in UKB. However, no statistically significant associations between frailty and the site-specific cancers were found in SALT. Frailty scores also provided limited added discriminative ability for cancers on top of age, sex, and traditional cancer risk factors. For any cancer, we performed a within-pair analysis in SALT and found that the association within DZ twins remained comparable to the population-level estimate, whereas the association within MZ twins was attenuated, indicating that genetic factors may in part explain the increased risk of incident cancer associated with frailty.

Given that both frailty and cancer are closely linked to aging and may share some of the underlying mechanisms such as chronic inflammation and immunosenescence [40–42], it is conceivable that there could be a bidirectional relationship between frailty and cancer. Nevertheless, prior research has primarily focused on the impact of frailty in older cancer patients [21]. Whether frailty may predict the risk of cancers in individuals without a history of cancer remain largely unexplored. The handful of studies to date mainly suggested no significant association between frailty scores and incident cancer [25–27]. For example, Aguayo et al. analyzed the association between 35 frailty scores, including the FI and FP, and incident self-reported cancer in 5,294 participants of the English Longitudinal Study of Ageing [26]. They found no significant associations in the fully adjusted models, and none of the frailty scores improved the C-statistic of the basic prediction model [26]. Klein et al. observed non-significant cross-sectional association between a functional frailty measure and self-reported cancer in 2,515 participants of the Beaver Dam Eye Study [25]. Petermann-Rocha et al. assessed the joint association of sarcopenia and FP on incident cancer in 341,668 UKB participants, but found no significant associations [27]. On the other hand, studies have suggested that some of the subcomponents of the FP may be associated with cancer risks. For example, a previous UKB study found that lower grip strength was associated with increased incidence of colorectal, lung, breast, and prostate cancers [43]. Similarly, low physical activity was found to be associated with higher risks of lung, breast, and colon cancers [44].

We can only speculate the reasons why we detected significant associations between frailty scores and incident cancers, while previous studies did not. The most likely explanations include the large sample and long follow-up. However, consistent with a previous study [26], we found that adding FI and FP to the model on any cancer resulted in no improvement of the predictive accuracy, while to the model on lung cancer resulted only in small improvement. It should also be noted that we found mixed results for lung cancer, with a strong association found in UKB but not in SALT. The discrepancy could be attributed to cohort or country-related differences other than the age of the participants or the follow-up time that were similar for both cohorts. We also found a reduced risk of melanoma with higher FI scores in UKB, which could be explained by the fact that frail individuals are usually less physically active and may spend less time outside and be exposed to ultraviolet radiation. Although we carefully controlled for several sun exposure and physical activity-related variables, there was a relatively high proportion of missing data in some of these variables, which may have led to misclassification. We were also unable to adjust for family history of melanoma as it was not available in UKB.

We performed a within-twin-pair analysis in SALT to assess if the observed association between frailty scores and any cancer may be influenced by shared genetic and/or environmental factors. The attenuation of the FI-cancer association within MZ twins implies that it could be attributable to genetic influences underlying both traits, instead of being causal. Intervening on frailty is therefore unlikely to result in significant improvement of cancer outcomes. However, this result should also be interpreted with caution. The attenuation of the association between the FI and any cancer in the within-pair analysis of MZ twins to < 1 is somewhat unexpected, given that the corresponding DZ estimate remained similar to population-level estimate in the full sample. In addition, when we performed the population-level analysis in the DZ and MZ twins, the estimates remained similar to the population-level estimate in the full sample, indicating that characteristics of the MZ twin sample per se are unlikely to explain the estimate of < 1. Of note, within-pair-estimates may be biased in the presence of confounding not shared by twins and measurement error in the exposure [45]. The CI was also wide for the within-pair estimate in MZ twins due to the relatively small sample size (n = 3,610 complete pairs). Hence, more studies are warranted to explore to which extent genetic factors account for the association between frailty and risk of cancer.

This study has strengths and limitations. The large sample consisting of two independent population-based cohorts enabled us to have enough statistical power to delineate the relationship between frailty and cancers that has thus far been understudied. The use of two different frailty measures also allowed us to compare and confirm the associations. However, UKB may not be representative to the general population due to healthy selection [31]. In particular, cancer incidence in UKB has shown to be lower than that in the general population, especially for older adults and women [31]. Besides, as we included mostly white participants, our results may not be generalizable to other populations. As in other observational studies, although we adjusted for several socio-economic and lifestyle factors, there may still be residual or unmeasured confounding. We also could not account for the stage or severity of cancer due to a lack of related information.

To conclude, this study is the largest so far to assess the predictive value of frailty on incident cancer, demonstrating that baseline frailty scores are significantly associated with a higher incidence of any cancer and lung cancer. Meanwhile, the associations may be confounded by genetic factors, and frailty scores provide only limited incremental discriminative ability over common cancer risk factors. Therefore, the currently available frailty scores may not be the optimal tools for cancer risk stratification at the population level.

## Supplementary Information

Below is the link to the electronic supplementary material.

Supplementary file1 (DOCX 1103 KB)

## Data Availability

The data used in this study are not publicly available due to ethical reasons. However, all bona fide researchers can apply to use the UK Biobank data for health-related research that is in the public interest (http://www.ukbiobank.ac.uk/register-apply). Data from the SALT study are available upon request from the Swedish Twin Registry for researchers who meet the criteria for access to confidential data (https://ki.se/en/research/the-swedish-twin-registry).
