# Supplementary material for: Can frailty scores predict the incidence of cancer? Results from two large population-based studies
Source: GeroScience. 2023 Mar 30;45(3):2051–64. doi: 10.1007/s11357-023-00783-9 (PMC10400738; doi:10.1007/s11357-023-00783-9)
Supplement: Supplementary file 1 — Supplementary file1 (DOCX 1103 KB) [file 11357_2023_783_MOESM1_ESM.docx]

**Can frailty scores predict the incidence of cancer? Results from two large population-based studies**

Jonathan K. L. Mak^1^*, Ralf Kuja-Halkola^1^, Yunzhang Wang^1,2^, Sara Hägg^1^, Juulia Jylhävä^1,3^

1. Department of Medical Epidemiology and Biostatistics, Karolinska Institutet, Stockholm, Sweden
2. Department of Clinical Sciences, Danderyd Hospital, Karolinska Institutet, Stockholm, Sweden
3. Faculty of Social Sciences (Health Sciences) and Gerontology Research Center (GEREC), University of Tampere, Tampere, Finland

* Corresponding author: Jonathan K. L. Mak

Department of Medical Epidemiology and Biostatistics, Karolinska Institutet, Nobels väg 12A, 171 77 Stockholm, Sweden

Email: jonathan.mak@ki.sei

Supplementary data

[Supplementary Table 1. List of the 49 and 44 items included in the frailty index in UK Biobank and SALT, respectively 2](#_Toc127720390)

[Supplementary Table 2. List of the five items used in construction of the frailty phenotype in UK Biobank 4](#_Toc127720391)

[Supplementary Table 3. List of the study variables in UK Biobank by frailty index categories 5](#_Toc127720392)

[Supplementary Table 4. List of the study variables in SALT by the frailty index categories. 7](#_Toc127720393)

[Supplementary Table 5. Associations between baseline frailty scores and the risk of lung cancer subtypes in UK Biobank (n = 453,144) 8](#_Toc127720394)

[Supplementary Table 6. Comparison of Harrell's C-statistics of Cox models 9](#_Toc127720395)

[Supplementary Table 7. Subgroup analysis for the associations between baseline frailty scores and the risk of any cancer in UK Biobank (n = 453,144) 10](#_Toc127720396)

[Supplementary Table 8. Subgroup analysis for the associations between baseline frailty index and the risk of any cancer in SALT (n = 36,888) 11](#_Toc127720397)

[Supplementary Table 9. Associations between baseline frailty index stripped from cancer-related items and the risk of any cancer in UK Biobank and SALT 12](#_Toc127720398)

[Supplementary Table 10. Associations between baseline frailty index stripped from lung cancer-related items and the risk of lung cancer in UK Biobank (n=453,144) 13](#_Toc127720399)

[Supplementary Table 11. Associations between baseline frailty scores by the frailty index and frailty phenotype and cancer incidence using complete data in UK Biobank and SALT 14](#_Toc127720400)

[Supplementary Figure 1. Flowchart for the selection of the UK Biobank and SALT analytical samples. 15](#_Toc127720401)

[Supplementary Figure 2. Box plot of the frailty index by the frailty phenotype scores in UK Biobank (n = 453,144) 16](#_Toc127720402)

[Supplementary Figure 3. Cumulative incidence of cancer over attained age, stratified by frailty status in UK Biobank and SALT 17](#_Toc127720403)

[Supplementary Figure 4. Estimated time-dependent hazard ratios for any cancer by frailty status in UK Biobank and SALT 18](#_Toc127720404)

# **Supplementary Table 1**. List of the 49 and 44 items included in the frailty index in UK Biobank and SALT, respectively

| **Category** | **Deficit item** | **UK Biobank** | **SALT** |
| --- | --- | --- | --- |
| Sensory | Glaucoma | Yes | - |
|  | Cataracts | Yes | - |
|  | Vision | - | Yes |
|  | Hearing | Yes | Yes |
|  | Buzzing in ears | - | Yes |
| Cranial | Migraine | Yes | Yes |
|  | Dizziness | - | Yes |
|  | Dental problems (ulcers, painful gums, bleeding gums, loose teeth, toothache, dentures) | Yes | - |
| Mental | Self-rated general health | Yes | Yes |
|  | Fatigue | Yes | - |
|  | Insomnia | Yes | - |
|  | Feeling depressed | Yes | Yes |
|  | Self-described nervous personality | Yes | - |
|  | Severe anxiety/ panic attacks | Yes | - |
|  | Feeling lonely | Yes | Yes |
|  | Feeling happy | - | Yes |
|  | Sense of misery | Yes | - |
|  | Health status prevents from doing things normally would like to do | - | Yes |
| Infirmity | Long-standing illness or disability | Yes | - |
|  | Falls in last year | Yes | - |
|  | Fractures/ broken bones | Yes | - |
|  | Physical handicap | - | Yes |
| Cardiometabolic | Diabetes | Yes | Yes |
|  | Heart attack/ myocardial infarction | Yes | Yes |
|  | Heart failure | - | Yes |
|  | Angina | Yes | Yes |
|  | Stroke | Yes | Yes |
|  | High blood pressure | Yes | Yes |
|  | Irregular cardiac rhythm/ atrial fibrillation | - | Yes |
|  | Hypothyroidism | Yes | - |
|  | Deep-vein thrombosis | Yes | Yes |
|  | Lipid disorder (e.g. high cholesterol, high triglycerides) | Yes | Yes |
|  | TIA attacks (temporary weakness, paralysis or reduction of sensibility) | - | Yes |
|  | Vascular spasm in legs (intermittent claudication) | - | Yes |

**Supplementary Table 1.** (continued)

| **Category** | **Deficit item** | **UK Biobank** | **SALT** |
| --- | --- | --- | --- |
| Respiratory | Wheezing | Yes | - |
|  | Pneumonia | Yes | - |
|  | Chronic lung disease (incl. chronic bronchitis/ emphysema) | Yes | Yes |
|  | Asthma | Yes | Yes |
|  | Recurrent periods of coughing | - | Yes |
| Musculoskeletal | Rheumatoid arthritis | Yes | Yes |
|  | Osteoarthritis | Yes | - |
|  | Osteoporosis | Yes | Yes |
|  | Gout | Yes | Yes |
| Immunological | Hay fever, allergic rhinitis or eczema | Yes | Yes |
|  | Psoriasis | Yes | - |
|  | Serious infections per year (other than respiratory) | - | Yes |
|  | Crohn's disease or ulcerative colitis | - | Yes |
| Cancer | Any cancer diagnosis (self-reported) | Yes | Yes |
|  | Multiple cancers (self-reported) | Yes | - |
| Pain | Chest pain | Yes | - |
|  | Head and/or neck pain | Yes | Yes |
|  | Back pain | Yes | Yes |
|  | Stomach/abdominal pain | Yes | - |
|  | Hip pain | Yes | Yes |
|  | Knee pain | Yes | Yes |
|  | Whole-body pain | Yes | - |
|  | Facial pain | Yes | - |
|  | Sciatica | Yes | Yes |
| Gastrointestinal | Gastric reflux | Yes | - |
|  | Hiatus hernia | Yes | - |
|  | Gall bladder problem, including gall stones | Yes | Yes |
|  | Diverticulitis | Yes | - |
|  | Stomach or intestine problems | - | Yes |
| Other | Glandular diseases (excl. goiter) | - | Yes |
|  | Goiter | - | Yes |
|  | Kidney disease | - | Yes |
|  | Liver disease (e.g. cirrhosis) | - | Yes |
|  | Recurring urinary tract problems | - | Yes |

# **Supplementary Table 2.** List of the five items used in construction of the frailty phenotype in UK Biobank

| **Frailty phenotype** | **Self-reported question/ measurement in UK Biobank** | **Scoring** |
| --- | --- | --- |
| Weight loss | “Compared with one year ago, has your weight changed?” | 1 = Yes, lost weight  0 = Other |
| Exhaustion | “Over the past two weeks, how often have you felt tired or had little energy?” | 1 = More than half the days or nearly every day  0 = Other |
| Slowness | “How would you describe your usual walking pace?” | 1 = Slow  0 = Other |
| Low physical activity | Physical activity questionnaire, categorized into 4 levels: | 1 = None, or light activity with a frequency of once per week or less  0 = Medium or heavy activity, or light activity more than once per week |
|  | 1. none – no physical activity in the last 4 weeks |  |
|  | 1. low – light DIY activity (e.g., pruning, watering the lawn) only in the last 4 weeks |  |
|  | 1. medium – heavy DIY activity (e.g., weeding, lawn mowing, carpentry and digging), walking for pleasure, or other exercises in the last 4 weeks |  |
|  | 1. high – strenuous sports in the last 4 weeks |  |
| Weakness | Measured grip strength (maximum value of either hand), stratified by sex and body mass index (BMI) | Women & BMI ≤23: 1 = grip strength ≤17 kg |
|  |  | Women & BMI >23–26: 1 = grip strength ≤17.3 kg |
|  |  | Women & BMI >26–29: 1 = grip strength ≤18 kg |
|  |  | Women & BMI >29: 1 = grip strength ≤21 kg |
|  |  | Men & BMI ≤24: 1 = grip strength ≤29 kg |
|  |  | Men & BMI >24–26: 1 = grip strength ≤30 kg |
|  |  | Men & BMI >26–28: 1 = grip strength ≤30 kg |
|  |  | Men & BMI >28: 1 = grip strength ≤32 kg |

# **Supplementary Table 3.** List of the study variables in UK Biobank by frailty index categories. Data are numbers (%) unless otherwise indicated.

| Variable | Relatively fit (n = 28,775) | Less fit (n = 173,252) | Least fit (n = 197,338) | Frail (n = 53,779) | *p*^a^ |
| --- | --- | --- | --- | --- | --- |
| Age at baseline, mean ± SD | 54.1 ± 8.0 | 55.6 ± 8.1 | 56.8 ± 8.1 | 57.9 ± 7.7 | <0.001 |
| Year of birth |  |  |  |  | <0.001 |
| 1930–1939 | 504 (1.8) | 4,724 (2.7) | 7,472 (3.8) | 2,572 (4.8) |  |
| 1940–1949 | 9,489 (33.0) | 68,528 (39.6) | 87,993 (44.6) | 26,248 (48.8) |  |
| 1950–1959 | 10,238 (35.6) | 58,112 (33.5) | 62,654 (31.7) | 16,676 (31.0) |  |
| ≥1960 | 8,544 (29.7) | 41,888 (24.2) | 39,219 (19.9) | 8,283 (15.4) |  |
| Men | 15,642 (54.4) | 83,609 (48.3) | 89,846 (45.5) | 22,972 (42.7) | <0.001 |
| Baseline assessment center | | | | | <0.001 |
| England | 25,157 (87.4) | 153,366 (88.5) | 175,634 (89.0) | 47,455 (88.2) |  |
| Wales | 1,128 (3.9) | 6,707 (3.9) | 8,351 (4.2) | 2,643 (4.9) |  |
| Scotland | 2,490 (8.7) | 13,179 (7.6) | 13,353 (6.8) | 3,681 (6.8) |  |
| Ethnic background |  |  |  |  | <0.001 |
| White | 27,205 (94.5) | 164,525 (95.0) | 185,728 (94.1) | 49,844 (92.7) |  |
| Asian | 640 (2.2) | 3,422 (2.0) | 4,605 (2.3) | 1,573 (2.9) |  |
| Black | 460 (1.6) | 2,454 (1.4) | 3,313 (1.7) | 1,079 (2.0) |  |
| Others | 355 (1.2) | 2,343 (1.4) | 3,039 (1.5) | 1,025 (1.9) |  |
| Missing | 115 (0.4) | 508 (0.3) | 653 (0.3) | 258 (0.5) |  |
| Body mass index |  |  |  |  | <0.001 |
| Underweight (<18.5) | 142 (0.5) | 919 (0.5) | 948 (0.5) | 273 (0.5) |  |
| Normal weight (18.5 to <25) | 12,422 (43.2) | 66,417 (38.3) | 58,538 (29.7) | 9,933 (18.5) |  |
| Overweight (25 to <30) | 12,479 (43.4) | 75,757 (43.7) | 85,230 (43.2) | 19,995 (37.2) |  |
| Obese (≥30) | 3,732 (13.0) | 30,159 (17.4) | 52,622 (26.7) | 23,578 (43.8) |  |
| Smoking status |  |  |  |  | <0.001 |
| Never | 19,013 (66.1) | 103,037 (59.5) | 103,579 (52.5) | 23,584 (43.9) |  |
| Previous | 7,548 (26.2) | 55,040 (31.8) | 71,420 (36.2) | 21,080 (39.2) |  |
| Current | 2,137 (7.4) | 14,712 (8.5) | 21,608 (10.9) | 8,837 (16.4) |  |
| Missing | 77 (0.3) | 463 (0.3) | 731 (0.4) | 278 (0.5) |  |
| Alcohol intake frequency |  |  |  |  | <0.001 |
| Less than weekly | 6,980 (24.3) | 43,459 (25.1) | 61,998 (31.4) | 24,550 (45.6) |  |
| Weekly | 21,778 (75.7) | 129,720 (74.9) | 135,180 (68.5) | 29,142 (54.2) |  |
| Missing | 17 (0.1) | 73 (0.0) | 160 (0.1) | 87 (0.2) |  |
| Education level^b^ |  |  |  |  | <0.001 |
| High | 12,504 (43.5) | 65,142 (37.6) | 60,193 (30.5) | 11,046 (20.5) |  |
| Intermediate | 13,611 (47.3) | 85,527 (49.4) | 99,430 (50.4) | 26,209 (48.7) |  |
| Low | 2,447 (8.5) | 21,205 (12.2) | 35,663 (18.1) | 15,732 (29.3) |  |
| Missing | 213 (0.7) | 1,378 (0.8) | 2,052 (1.0) | 792 (1.5) |  |
| Deprivation index quintiles^c^ |  |  |  |  | <0.001 |
| 1 (least deprived) | 6,671 (23.2) | 38,719 (22.3) | 38,118 (19.3) | 7,032 (13.1) |  |
| 2 | 6,477 (22.5) | 37,029 (21.4) | 39,092 (19.8) | 8,032 (14.9) |  |
| 3 | 5,968 (20.7) | 35,703 (20.6) | 39,355 (19.9) | 9,361 (17.4) |  |
| 4 | 5,382 (18.7) | 33,818 (19.5) | 39,870 (20.2) | 11,446 (21.3) |  |
| 5 (most deprived) | 4,252 (14.8) | 27,776 (16.0) | 40,653 (20.6) | 17,829 (33.2) |  |
| Missing | 25 (0.1) | 207 (0.1) | 250 (0.1) | 79 (0.1) |  |
| Family history of breast cancer^d^ | 2,852 (9.9) | 17,602 (10.2) | 20,413 (10.3) | 5,566 (10.3) | 0.06 |
| Family history of prostate cancer^d^ | 2,121 (7.4) | 13166 (7.6) | 15,036 (7.6) | 4,276 (8.0) | 0.013 |
| Family history of lung cancer^d^ | 2,924 (10.2) | 19,489 (11.2) | 24,902 (12.6) | 8,294 (15.4) | <0.001 |
| Family history of colorectal cancer^d^ | 2,894 (10.1) | 17,753 (10.2) | 21,742 (11.0) | 6,572 (12.2) | <0.001 |
| Physical activity level^e^ |  |  |  |  | <0.001 |
| Low | 4,561 (15.9) | 32,646 (18.8) | 45,430 (23.0) | 17,174 (31.9) |  |
| Moderate | 10,879 (37.8) | 67,556 (39.0) | 76,677 (38.9) | 18,717 (34.8) |  |
| High | 12,659 (44.0) | 68,298 (39.4) | 68,353 (34.6) | 15,132 (28.1) |  |
| Missing | 676 (2.3) | 4,752 (2.7) | 6,878 (3.5) | 2,756 (5.1) |  |
| Time spent outdoors during summer^f^ | | | | | <0.001 |
| <1h/day | 980 (3.4) | 6,173 (3.6) | 8,291 (4.2) | 3,283 (6.1) |  |
| 1-2h/day | 9,216 (32.0) | 53,375 (30.8) | 57,086 (28.9) | 13,556 (25.2) |  |
| 3-5h/day | 11,982 (41.6) | 72,944 (42.1) | 81,991 (41.5) | 21,470 (39.9) |  |
| >5h/day | 5,395 (18.7) | 32,366 (18.7) | 38,522 (19.5) | 11,228 (20.9) |  |
| Missing | 1,202 (4.2) | 8,398 (4.8) | 11,450 (5.8) | 4,246 (7.9) |  |

**Supplementary Table 3.** (continued)

| Variable | Relatively fit (n = 28,775) | Less fit (n = 173,252) | Least fit (n = 197,338) | Frail (n = 53,779) | *p*^a^ |
| --- | --- | --- | --- | --- | --- |
| Use of sun/UV protection^g^ |  |  |  |  | <0.001 |
| Never/rarely | 2,654 (9.2) | 15,360 (8.9) | 20,705 (10.5) | 7,836 (14.6) |  |
| Sometimes | 9,451 (32.8) | 57,563 (33.2) | 66,581 (33.7) | 18,104 (33.7) |  |
| Most of the time | 10,577 (36.8) | 64,149 (37.0) | 69,169 (35.1) | 16,095 (29.9) |  |
| Always | 5,962 (20.7) | 35,346 (20.4) | 39,470 (20.0) | 10,869 (20.2) |  |
| Do not go out in sunshine | 91 (0.3) | 661 (0.4) | 1,131 (0.6) | 745 (1.4) |  |
| Missing | 40 (0.1) | 173 (0.1) | 282 (0.1) | 130 (0.2) |  |
| Sunburn during childhood^h^ |  |  |  |  | <0.001 |
| No | 12,979 (45.1) | 72,812 (42.0) | 78,480 (39.8) | 21,267 (39.5) |  |
| Yes | 9,796 (34.0) | 59,884 (34.6) | 69,150 (35.0) | 18,664 (34.7) |  |
| Missing | 6,000 (20.9) | 40,556 (23.4) | 49,708 (25.2) | 13,848 (25.7) |  |
| Solarium/sunlamp use^i^ |  |  |  |  | <0.001 |
| No | 27,493 (95.5) | 164,663 (95.0) | 185,885 (94.2) | 50,196 (93.3) |  |
| Yes | 1,076 (3.7) | 7,355 (4.2) | 9,326 (4.7) | 2,559 (4.8) |  |
| Missing | 206 (0.7) | 1,234 (0.7) | 2,127 (1.1) | 1,024 (1.9) |  |
| Ease of skin tanning^j^ |  |  |  |  | <0.001 |
| Get very tanned | 6,014 (20.9) | 35,733 (20.6) | 41,797 (21.2) | 12,269 (22.8) |  |
| Get moderately tanned | 12,149 (42.2) | 70,866 (40.9) | 75,584 (38.3) | 17,762 (33.0) |  |
| Get mildly or occasionally tanned | 5,956 (20.7) | 36,002 (20.8) | 40,831 (20.7) | 10,811 (20.1) |  |
| Never tan, only burn | 3,937 (13.7) | 26,311 (15.2) | 33,871 (17.2) | 11,184 (20.8) |  |
| Missing | 719 (2.5) | 4,340 (2.5) | 5,255 (2.7) | 1,753 (3.3) |  |
| Skin color^k^ |  |  |  |  | <0.001 |
| Black, brown | 1,049 (3.6) | 5,678 (3.3) | 7,675 (3.9) | 2,565 (4.8) |  |
| Light, dark olive | 6,218 (21.6) | 36,044 (20.8) | 39,334 (19.9) | 10,195 (19.0) |  |
| Fair | 19,402 (67.4) | 117,933 (68.1) | 132,018 (66.9) | 34,502 (64.2) |  |
| Very fair | 1,734 (6.0) | 11,533 (6.7) | 15,531 (7.9) | 5,484 (10.2) |  |
| Missing | 372 (1.3) | 2,064 (1.2) | 2,780 (1.4) | 1,033 (1.9) |  |
| Hair color^l^ |  |  |  |  | <0.001 |
| Black, dark brown, other | 13,794 (47.9) | 80,767 (46.6) | 91,889 (46.6) | 25,339 (47.1) |  |
| Light brown | 10,888 (37.8) | 66,731 (38.5) | 75,954 (38.5) | 20,036 (37.3) |  |
| Blonde, red | 4,044 (14.1) | 25,475 (14.7) | 29,097 (14.7) | 8,241 (15.3) |  |
| Missing | 49 (0.2) | 279 (0.2) | 398 (0.2) | 163 (0.3) |  |

1. *P*-values for comparison between frailty index categories were based on *t*-tests for continuous variables and *χ*^2^ tests for categorical variables.
2. Education was assessed by the highest self-reported qualification: low (no relevant qualifications); intermediate (A levels, O levels/GCSEs, CSEs, NVQ/HND/HNC, other professional qualifications); high (college or university degree).
3. Townsend deprivation index was derived from national census data regarding unemployment, car ownership, home ownership, and household overcrowding. A higher score indicates a higher level of socioeconomic deprivation.
4. Family history of cancer was defined by any self-reported cancers in father, mother or siblings.
5. Physical activity was assessed by the self-reported International Physical Activity Questionnaire (IPAQ) and was categorized into low, moderate, high according to the protocol.
6. Time spent outdoors during summer was assessed by the question “in a typical DAY in summer, how many hours do you spend outdoors?”
7. Use of UV protection was assessed by the question “do you wear sun protection (e.g. sunscreen lotion, hat) when you spend time outdoors in the summer?”
8. Childhood sunburns were assessed by the question “before the age of 15, how many times did you suffer sunburn that was painful for at least 2 days or caused blistering?”
9. Solarium/sunlamp use was assessed by the question “how many times a year would you use a solarium or sunlamp?”, and was categorized into no (0 time a year) and yes (1 or more time a year).
10. Ease of skin tanning was assessed by the question “what would happen to your skin if it was repeatedly exposed to bright sunlight without any protection?”
11. Skin color was assessed by the question “what best describes the color of your skin without tanning?”
12. Hair color was assessed by the question “what best describes your natural hair color? (If your hair color is grey, the color before you went grey)”

# **Supplementary Table 4.** List of the study variables in SALT by the frailty index categories. Data are numbers (%) unless otherwise indicated.

| Variable | Relatively fit (n = 3,805) | Less fit (n = 14,090) | Least fit (n = 13,665) | Frail (n = 5,328) | *p*^a^ |
| --- | --- | --- | --- | --- | --- |
| Age at baseline, mean ± SD | 53.9 ± 7.7 | 55.2 ± 7.8 | 56.6 ± 8.1 | 58.3 ± 8.1 | <0.001 |
| Year of birth |  |  |  |  | <0.001 |
| <1930 | 174 (4.6) | 887 (6.3) | 1,247 (9.1) | 686 (12.9) |  |
| 1930–1939 | 689 (18.1) | 2,976 (21.1) | 3,450 (25.2) | 1,539 (28.9) |  |
| 1940–1949 | 1,384 (36.4) | 5,398 (38.3) | 5,154 (37.7) | 2,020 (37.9) |  |
| 1950-1959 | 1,558 (40.9) | 4,829 (34.3) | 3,814 (27.9) | 1,083 (20.3) |  |
| Men | 2088 (54.9) | 7421 (52.7) | 6315 (46.2) | 1855 (34.8) | <0.001 |
| Body mass index |  |  |  |  | <0.001 |
| Underweight (<18.5) | 44 (1.2) | 154 (1.1) | 146 (1.1) | 75 (1.4) |  |
| Normal weight (18.5 to <25) | 2,246 (59.0) | 7,807 (55.4) | 6,767 (49.5) | 2,202 (41.3) |  |
| Overweight (25 to <30) | 1,306 (34.3) | 5,098 (36.2) | 5,236 (38.3) | 2,129 (40.0) |  |
| Obese (≥30) | 152 (4.0) | 837 (5.9) | 1,257 (9.2) | 802 (15.1) |  |
| Missing | 57 (1.5) | 194 (1.4) | 259 (1.9) | 120 (2.3) |  |
| Smoking status |  |  |  |  | <0.001 |
| Never | 1,697 (44.6) | 5,819 (41.3) | 5,125 (37.5) | 1,871 (35.1) |  |
| Previous | 1,318 (34.6) | 5,355 (38.0) | 5,455 (39.9) | 2,078 (39.0) |  |
| Current | 782 (20.6) | 2,881 (20.4) | 3,029 (22.2) | 1,341 (25.2) |  |
| Missing | 8 (0.2) | 35 (0.2) | 56 (0.4) | 38 (0.7) |  |
| Alcohol intake frequency |  |  |  |  | <0.001 |
| Less than weekly | 821 (21.6) | 3,421 (24.3) | 4,045 (29.6) | 2,096 (39.3) |  |
| Weekly | 2,789 (73.3) | 9,942 (70.6) | 8,804 (64.4) | 2,786 (52.3) |  |
| Missing | 195 (5.1) | 727 (5.2) | 816 (6.0) | 446 (8.4) |  |
| Education level^b^ |  |  |  |  | <0.001 |
| High | 1,218 (32.0) | 4,193 (29.8) | 3,217 (23.5) | 989 (18.6) |  |
| Intermediate | 1,912 (50.2) | 6,828 (48.5) | 6,677 (48.9) | 2,560 (48.0) |  |
| Low | 662 (17.4) | 3,016 (21.4) | 3,703 (27.1) | 1,736 (32.6) |  |
| Missing | 13 (0.3) | 53 (0.4) | 68 (0.5) | 43 (0.8) |  |

Abbreviations: SALT, Screening Across the Lifespan Twin Study; SD, standard deviation

1. *P*-values for comparison between frailty index categories were based on *t*-tests for continuous variables and *χ*^2^ tests for categorical variables.
2. Education level in SALT was defined by years of completed education: low (<9 years); intermediate (9–12 years); high (>12 years)

# **Supplementary Table 5.** Associations between baseline frailty scores and the risk of lung cancer subtypes in UK Biobank (n = 453,144). Data are hazard ratios (95% confidence intervals) unless otherwise indicated.

| Lung cancer subtypes^a^ | **Frailty index** | | | | | **Frailty phenotype** | | |
| --- | --- | --- | --- | --- | --- | --- | --- | --- |
|  | Relatively fit | Less fit | Least fit | Frail | Per 10% increase | Non-frail | Pre-frail | Frail |
| **Adenocarcinoma** | | | | | | | | |
| Incidence per 100,000 person-years | 7.6 | 22.6 | 32.4 | 53.3 | - | 24.1 | 35.8 | 50.3 |
| Age- and sex-adjusted model^b^ | 1  (ref.) | 2.62  (1.74, 3.96)* | 3.43  (2.28, 5.15)* | 5.25  (3.46, 7.96)* | 1.46  (1.37, 1.55)* | 1  (ref.) | 1.44  (1.29, 1.60)* | 1.89  (1.51, 2.36)* |
| Multivariable model^c^ | 1  (ref.) | 2.34  (1.79, 4.05)* | 2.69  (1.79, 4.05)* | 3.45  (2.27, 5.25)* | 1.26  (1.18, 1.35)* | 1  (ref.) | 1.28  (1.15, 1.43)* | 1.32  (1.05, 1.67) |
| Lung cancer-specific model^d^ | 1  (ref.) | 2.34  (1.55, 3.52)* | 2.68  (1.78, 4.03)* | 3.41  (2.24, 5.20)* | 1.25  (1.17, 1.34)* | 1  (ref.) | 1.27  (1.14, 1.42)* | 1.31  (1.04, 1.65) |
| **Squamous cell carcinoma** | | | | | | | | |
| Incidence per 100,000 person-years | 4.7 | 7.3 | 15.0 | 38.0 | - | 9.4 | 17.8 | 51.5 |
| Age- and sex-adjusted model^b^ | 1  (ref.) | 1.41  (0.83, 2.41) | 2.68  (1.60, 4.51)* | 6.51  (3.86, 11.0)* | 2.02  (1.87, 2.19)* | 1  (ref.) | 1.93  (1.64, 2.27)* | 5.45  (4.28, 6.94)* |
| Multivariable model^c^ | 1  (ref.) | 1.15  (0.67, 1.96) | 1.68  (1.00, 2.83) | 2.70  (1.59, 4.59)* | 1.47  (1.35, 1.61)* | 1  (ref.) | 1.43  (1.21, 1.69)* | 2.39  (1.85, 3.09)* |
| Lung cancer-specific model^d^ | 1  (ref.) | 1.15  (0.67, 1.96) | 1.67  (0.99, 2.80) | 2.65  (1.56, 4.51)* | 1.46  (1.34, 1.59)* | 1  (ref.) | 1.42  (1.20, 1.67)* | 2.35  (1.82, 3.04)* |

1. Adenocarcinoma was defined by the ICD-O-3 codes 8140, 8211, 8230, 8231, 8255–8260, 8323, 8480–8490, 8550, 8551, 8570–8574, or 8576. Squamous cell carcinoma was defined by the ICD-O-3 codes of 8050–8078, 8083, or 8084. Other lung cancer subtypes were not analyzed due to a lack of statistical power.
2. Age- and sex-adjusted model: adjusted for age (time scale), birth year (1930–1939, 1940–1949, 1950–1959, ≥1960), and sex.
3. Multivariable model: age- and sex-adjusted model + baseline assessment center, ethnic background, body mass index, smoking status, alcohol consumption, education level, deprivation index quintiles.
4. Lung cancer-specific model: multivariable model + family history of lung cancer (no, yes).

* Significant after Bonferroni adjustment at *p*<.005 (i.e., .05/10, considering 2 frailty measures × 5 cancers)

# **Supplementary Table 6.** Comparison of Harrell's C-statistics of Cox models

| Outcome | Model | **UK Biobank (n = 453,144)** | | **SALT (n = 36,888)** | |
| --- | --- | --- | --- | --- | --- |
|  |  | Harrell’s C | 95% CI^a^ | Harrell’s C | 95% CI^a^ |
| Any cancer | M1: Age, birth year, sex^b^ | 0.533 | (0.531, 0.535) | 0.526 | (0.519, 0.536) |
|  | M2: Age, birth year, sex, common risk factors^c^ | 0.556 | (0.554, 0.559) | 0.553 | (0.547, 0.564) |
|  | M3: Age, birth year, sex, common risk factors, FI score | 0.557 | (0.555, 0.560) | 0.557 | (0.550, 0.568) |
|  | M4: Age, birth year, sex, common risk factors, FP score^d^ | 0.557 | (0.555, 0.560) | - | - |
| Breast cancer | M1: Age, birth year^b^ | 0.503 | (0.501, 0.508) | 0.510 | (0.503, 0.527) |
|  | M2: Age, birth year, common risk factors^c^ | 0.555 | (0.551, 0.563) | 0.547 | (0.536, 0.578) |
|  | M3: Age, birth year, common risk factors, FI score | 0.555 | (0.551, 0.564) | 0.554 | (0.544, 0.586) |
|  | M4: Age, birth year, common risk factors, FP score^d^ | 0.555 | (0.551, 0.564) | - | - |
| Prostate cancer | M1: Age, birth year^b^ | 0.503 | (0.501, 0.507) | 0.509 | (0.503, 0.525) |
|  | M2: Age, birth year, common risk factors^c^ | 0.564 | (0.559, 0.571) | 0.550 | (0.540, 0.575) |
|  | M3: Age, birth year, common risk factors, FI score | 0.564 | (0.560, 0.571) | 0.556 | (0.545, 0.581) |
|  | M4: Age, birth year, common risk factors, FP score^d^ | 0.564 | (0.560, 0.571) | - | - |
| Lung cancer | M1: Age, birth year, sex^b^ | 0.534 | (0.526, 0.543) | 0.528 | (0.495, 0.564) |
|  | M2: Age, birth year, sex, common risk factors^c^ | 0.779 | (0.771, 0.789) | 0.758 | (0.734, 0.790) |
|  | M3: Age, birth year, sex, common risk factors, FI score | 0.784 | (0.776, 0.794) | 0.758 | (0.736, 0.791) |
|  | M4: Age, birth year, sex, common risk factors, FP score^d^ | 0.782 | (0.774, 0.791) | - | - |
| Colorectal cancer | M1: Age, birth year, sex^b^ | 0.564 | (0.557, 0.572) | 0.537 | (0.513, 0.568) |
|  | M2: Age, birth year, sex, common risk factors^c^ | 0.589 | (0.584, 0.599) | 0.555 | (0.546, 0.604) |
|  | M3: Age, birth year, sex, common risk factors, FI score | 0.590 | (0.584, 0.600) | 0.562 | (0.551, 0.608) |
|  | M4: Age, birth year, sex, common risk factors, FP score^d^ | 0.589 | (0.584, 0.600) | - | - |
| Melanoma | M1: Age, birth year, sex^b^ | 0.526 | (0.518, 0.539) | 0.538 | (0.512, 0.586) |
|  | M2: Age, birth year, sex, common risk factors^c^ | 0.641 | (0.635, 0.657) | 0.594 | (0.580, 0.649) |
|  | M3: Age, birth year, sex, common risk factors, FI score | 0.642 | (0.636, 0.658) | 0.593 | (0.581, 0.650) |
|  | M4: Age, birth year, sex, common risk factors, FP score^d^ | 0.641 | (0.636, 0.657) | - | - |

Abbreviations: CI, confidence interval; FI, frailty index; FP, frailty phenotype; SALT, Screening Across the Lifespan Twin Study.

1. 95% CIs of the Harrell's C-statistics were computed through 1000-times bootstrapping resampling.
2. Model M1 included age (time scale), birth year (1930–1939, 1940–1949, 1950–1959, ≥1960), and sex (except for breast cancer and prostate cancer).
3. Model M2: M1 + baseline assessment center, ethnic background, body mass index, smoking status, alcohol consumption, education level, deprivation index quintiles, and the cancer-specific covariates.
4. FP was not available in SALT.

# **Supplementary Table 7.** Subgroup analysis for the associations between baseline frailty scores and the risk of any cancer in UK Biobank (n = 453,144). Data are hazard ratios (95% confidence intervals) unless otherwise indicated.^a^

| Subgroup | **Frailty index** | | | | | | **Frailty phenotype** | | | |
| --- | --- | --- | --- | --- | --- | --- | --- | --- | --- | --- |
|  | Relatively fit | Less fit | Least fit | Frail | Per 10% increase | *P*_interaction_^b^ | Non-frail | Pre-frail | Frail | *P*_interaction_^b^ |
| **Age at baseline** | | | | | | | | | | |
| <60 years  (n = 262,153) | 1  (ref.) | 1.10  (1.04 1.17)* | 1.13  (1.06, 1.19)* | 1.25  (1.17, 1.34)* | 1.07  (1.05, 1.09)* | 0.20 | 1 (ref.) | 1.03  (1.00, 1.06) | 1.15  (1.07, 1.24)* | 0.40 |
| ≥60 years  (n = 190,091) | 1  (ref.) | 1.07  (1.01, 1.14) | 1.12  (1.06, 1.19)* | 1.19  (1.12, 1.27)* | 1.07  (1.05, 1.08)* |  | 1 (ref.) | 1.05  (1.03, 1.08)* | 1.16  (1.09, 1.22)* |  |
| **Sex** | | | | | | | | | | |
| Women  (n = 241,075) | 1  (ref.) | 1.06  (1.00, 1.13) | 1.07  (1.01, 1.14) | 1.16  (1.09, 1.24)* | 1.05  (1.03, 1.07)* | <0.001 | 1 (ref.) | 1.04  (1.01, 1.06) | 1.13  (1.06, 1.20)* | 0.040 |
| Men  (n = 212,069) | 1  (ref.) | 1.11  (1.05, 1.17)* | 1.18  (1.12, 1.24)* | 1.27  (1.19, 1.35)* | 1.09  (1.07, 1.10)* |  | 1 (ref.) | 1.05  (1.03, 1.08)* | 1.17  (1.10, 1.25)* |  |
| **BMI** | | | | | | | | | | |
| <25 kg/m^2^  (n = 149,592) | 1  (ref.) | 1.07  (1.01, 1.14) | 1.10  (1.03, 1.17) | 1.18  (1.09, 1.28)* | 1.07  (1.04, 1.10)* | 0.71 | 1 (ref.) | 1.05  (1.01, 1.09) | 1.40  (1.27, 1.56)* | 0.018 |
| ≥25 kg/m^2^  (n = 303,552) | 1  (ref.) | 1.10  (1.05, 1.16)* | 1.16  (1.10, 1.22)* | 1.24  (1.17, 1.31)* | 1.07  (1.05, 1.08)* |  | 1 (ref.) | 1.04  (1.02, 1.06)* | 1.11  (1.06, 1.17)* |  |
| **Smoking status** | | | | | | | | | | |
| Non-smokers  (n = 249,213) | 1  (ref.) | 1.08  (1.02, 1.14) | 1.11  (1.05, 1.17)* | 1.13  (1.07, 1.21)* | 1.05  (1.03, 1.06)* | <0.001 | 1 (ref.) | 1.01  (0.99, 1.04) | 1.09  (1.01, 1.17) | <0.001 |
| Ever-smokers  (n = 202,382) | 1  (ref.) | 1.12  (1.05, 1.19)* | 1.18  (1.10, 1.25)* | 1.30  (1.21, 1.39)* | 1.09  (1.07, 1.10)* |  | 1 (ref.) | 1.08  (1.05, 1.11)* | 1.20  (1.13, 1.27)* |  |

1. All models were adjusted for age (time scale), birth year, sex, baseline assessment center, ethnic background, body mass index, smoking status, alcohol consumption, education level, and deprivation index quintiles, except when the variable was used as the subgroup.
2. *P*-values for the multiplicative interaction terms between the continuous frailty scores and the subgroup indicator.

* Significant after Bonferroni adjustment at *p*<.005 (i.e., .05/10, considering 2 frailty measures × 5 cancers)

# **Supplementary Table 8.** Subgroup analysis for the associations between baseline frailty index and the risk of any cancer in SALT (n = 36,888). Data are hazard ratios (95% confidence intervals) unless otherwise indicated.^a^

| Subgroup | **Frailty index** | | | | | |
| --- | --- | --- | --- | --- | --- | --- |
|  | Relatively fit | Less fit | Least fit | Frail | Per 10% increase | *P*_interaction_^b^ |
| **Age at baseline** | | | | | | |
| <60 years (n = 25,420) | 1 (ref.) | 1.05 (0.90, 1.22) | 1.09 (0.94, 1.27) | 1.22 (1.02, 1.46) | 1.05 (1.00, 1.11) | 0.68 |
| ≥60 years (n = 11,468) | 1 (ref.) | 1.13 (0.94, 1.34) | 1.19 (1.00, 1.42) | 1.41 (1.17, 1.71)* | 1.10 (1.05, 1.15)* |  |
| **Sex** | | | | | | |
| Women (n = 19,209) | 1 (ref.) | 1.06 (0.89, 1.27) | 1.12 (0.94, 1.34) | 1.29 (1.06, 1.56) | 1.07 (1.02, 1.12) | 0.33 |
| Men (n = 17,679) | 1 (ref.) | 1.08 (0.93, 1.25) | 1.13 (0.97, 1.31) | 1.32 (1.11, 1.57)* | 1.08 (1.03, 1.14)* |  |
| **BMI** | | | | | | |
| <25 kg/m^2^ (n = 19,441) | 1 (ref.) | 1.10 (0.94, 1.28) | 1.16 (0.99, 1.35) | 1.45 (1.22, 1.74)* | 1.10 (1.05, 1.16)* | 0.30 |
| ≥25 kg/m^2^ (n = 16,817) | 1 (ref.) | 1.06 (0.89, 1.26) | 1.12 (0.94, 1.33) | 1.16 (0.96, 1.40) | 1.05 (1.00, 1.10) |  |
| **Smoking status** | | | | | | |
| Non-smokers (n = 14,512) | 1 (ref.) | 0.97 (0.82, 1.15) | 1.02 (0.86, 1.22) | 1.28 (1.04, 1.57) | 1.10 (1.03, 1.17)* | 0.69 |
| Ever-smokers (n = 22,239) | 1 (ref.) | 1.16 (1.00, 1.36) | 1.22 (1.05, 1.42) | 1.33 (1.12, 1.57)* | 1.06 (1.02, 1.11) |  |

Abbreviation: SALT, Screening Across the Lifespan Twin Study

1. All models were adjusted for age (time scale), birth year, sex, body mass index, smoking status, alcohol consumption, and education level, except when the variable was used as the subgroup.
2. *P*-values for the multiplicative interaction terms between the continuous frailty score and the subgroup indicator.

* Significant after Bonferroni adjustment at *p*<.005 (i.e., .05/10, considering 2 frailty measures × 5 cancers)

# **Supplementary Table 9.** Associations between baseline frailty index stripped from cancer-related items and the risk of any cancer in UK Biobank and SALT. Data are hazard ratios (95% confidence intervals) unless otherwise indicated

|  | **Frailty index with self-reported cancer items removed^a^** | | | | |
| --- | --- | --- | --- | --- | --- |
|  | Relatively fit | Less fit | Least fit | Frail | Per 10% increase |
| **UK Biobank (n = 453,144)** | | | | | |
| Incidence per 100,000 person-years | 887.4 | 1038.3 | 1170.2 | 1347.2 | - |
| Age- and sex-adjusted model^b^ | 1 (ref.) | 1.10 (1.06, 1.15)* | 1.17 (1.12, 1.21)* | 1.29 (1.23, 1.34)* | 1.09 (1.08, 1.10)* |
| Multivariable model^c^ | 1 (ref.) | 1.08 (1.04, 1.13)* | 1.12 (1.08, 1.17)* | 1.20 (1.15, 1.25)* | 1.06 (1.05, 1.08)* |
| **SALT (n = 36,888)** | | | | | |
| Incidence per 100,000 person-years | 919.1 | 1057.2 | 1201.6 | 1447.5 | - |
| Age- and sex-adjusted model^b^ | 1 (ref.) | 1.07 (0.96, 1.20) | 1.13 (1.01, 1.27) | 1.30 (1.15, 1.48)* | 1.08 (1.04, 1.12)* |
| Multivariable model^d^ | 1 (ref.) | 1.06 (0.95, 1.19) | 1.12 (1.00, 1.25) | 1.27 (1.12, 1.45)* | 1.07 (1.04, 1.11)* |

Abbreviations: SALT, Screening Across the Lifespan Twin Study

1. A 47-item frailty index was used in UK Biobank (removed “any cancer diagnosis”, “multiple cancers”); and a 43-item frailty index was used in SALT (removed “any cancer diagnosis”).
2. Age- and sex-adjusted model: adjusted for age (time scale), birth year, and sex
3. Multivariable model in UK Biobank: age- and sex-adjusted model + baseline assessment center, ethnic background, body mass index, smoking status, alcohol consumption, education level, deprivation index quintiles.
4. Multivariable model in SALT: age- and sex-adjusted model + body mass index, smoking status, alcohol consumption, and education level.

* Significant after Bonferroni adjustment at *p*<.005 (i.e., .05/10, considering 2 frailty measures × 5 cancers)

# **Supplementary Table 10.** Associations between baseline frailty index stripped from lung cancer-related items and the risk of lung cancer in UK Biobank (n=453,144). Data are hazard ratios (95% confidence intervals) unless otherwise indicated.

|  | **Frailty index with lung cancer-related items removed^a^** | | | | |
| --- | --- | --- | --- | --- | --- |
|  | Relatively fit | Less fit | Least fit | Frail | Per 10% increase |
| Incidence per 100,000 person-years | 28.7 | 50.5 | 78.3 | 145.0 | - |
| Age- and sex-adjusted model^b^ | 1 (ref.) | 1.61 (1.31, 1.97)* | 2.25 (1.84, 2.74)* | 3.87 (3.16, 4.75)* | 1.55 (1.50, 1.61)* |
| Multivariable model^c^ | 1 (ref.) | 1.41 (1.15, 1.74)* | 1.70 (1.39, 2.08)* | 2.31 (1.87, 2.84)* | 1.29 (1.24, 1.34)* |
| Lung cancer-specific model^d^ | 1 (ref.) | 1.41 (1.15, 1.73)* | 1.69 (1.39, 2.07)* | 2.28 (1.85, 2.80)* | 1.28 (1.23, 1.33)* |

1. A 42-item frailty index was used in UK Biobank (removed “wheezing”, “pneumonia”, “chronic lung disease”, “asthma”, “chest pain”, “any cancer diagnosis”, “multiple cancers”).
2. Age- and sex-adjusted model: adjusted for age (time scale), birth year, and sex
3. Multivariable model: age- and sex-adjusted model + baseline assessment center, ethnic background, body mass index, smoking status, alcohol consumption, education level, deprivation index quintiles.
4. Lung cancer-specific model: multivariable model + family history of lung cancer.

* Significant after Bonferroni adjustment at *p*<.005 (i.e., .05/10, considering 2 frailty measures × 5 cancers)

# **Supplementary Table 11.** Associations between baseline frailty scores by the frailty index and frailty phenotype and cancer incidence using complete data in UK Biobank and SALT^a^. Data are hazard ratios (95% confidence intervals) unless otherwise indicated.

| Cancer site | **Frailty index** | | | | | **Frailty phenotype** | | |
| --- | --- | --- | --- | --- | --- | --- | --- | --- |
|  | Relatively fit | Less fit | Least fit | Frail | Per 10% increase | Non-frail | Pre-frail | Frail |
| **Any cancer** | | | | | | | | |
| UKB, multivariable model  (n = 445,019)^b^ | 1 (ref.) | 1.09 (1.05, 1.14)* | 1.13 (1.09, 1.18)* | 1.22 (1.16, 1.27)* | 1.07 (1.06, 1.08)* | 1 (ref.) | 1.05 (1.03, 1.07)* | 1.15 (1.10, 1.20)* |
| SALT, multivariable model  (n = 34,222)^c^ | 1 (ref.) | 1.09 (0.97, 1.23) | 1.15 (1.02, 1.30) | 1.28 (1.12, 1.47)* | 1.06 (1.03, 1.11)* | - | - | - |
| **Breast cancer in women** | | | | | | | | |
| UKB, breast cancer-specific model  (n = 237,033)^d^ | 1 (ref.) | 1.03 (0.93, 1.14) | 1.00 (0.90, 1.11) | 1.04 (0.93, 1.17) | 1.00 (0.97, 1.04) | 1 (ref.) | 1.02 (0.97, 1.07) | 0.97 (0.96, 1.09) |
| SALT, multivariable model  (n = 17,353)^c^ | 1 (ref.) | 1.15 (0.84, 1.59) | 1.20 (0.88, 1.64) | 1.44 (1.03, 2.03) | 1.09 (1.00, 1.18) | - | - | - |
| **Prostate cancer in men** | | | | | | | | |
| UKB, prostate cancer-specific model  (n = 207,986)^e^ | 1 (ref.) | 1.11 (1.02, 1.21) | 1.10 (1.01, 1.20) | 1.05 (0.95, 1.16) | 1.00 (0.97, 1.03) | 1 (ref.) | 0.97 (0.93, 1.01) | 0.87 (0.76, 0.98) |
| SALT, multivariable model  (n = 16,869)^c^ | 1 (ref.) | 1.22 (0.96, 1.56) | 1.44 (1.13, 1.84)* | 1.46 (1.10, 1.95) | 1.10 (1.02, 1.19) | - | - | - |
| **Lung cancer** | | | | | | | | |
| UKB, lung cancer-specific model  (n = 445,019)^f^ | 1 (ref.) | 1.44 (1.15, 1.80)* | 1.81 (1.45, 2.26)* | 2.63 (2.09, 3.31)* | 1.36 (1.30, 1.41)* | 1 (ref.) | 1.32 (1.23, 1.41)* | 1.90 (1.68, 2.16)* |
| SALT, multivariable model  (n = 34,222)^c^ | 1 (ref.) | 0.74 (0.49, 1.10) | 0.87 (0.59, 1.29) | 0.91 (0.59, 1.41) | 1.06 (0.93, 1.21) | - | - | - |
| **Colorectal cancer** | | | | | | | | |
| UKB, colorectal cancer-specific model  (n = 445,019)^g^ | 1 (ref.) | 1.12 (0.99, 1.27) | 1.12 (0.99, 1.28) | 1.10 (0.95, 1.27) | 0.99 (0.95, 1.03) | 1 (ref.) | 1.06 (1.01, 1.13) | 1.02 (0.87, 1.18) |
| SALT, multivariable model  (n = 34,222)^c^ | 1 (ref.) | 0.99 (0.68, 1.44) | 0.95 (0.65, 1.39) | 0.87 (0.56, 1.35) | 0.90 (0.79, 1.02) | - | - | - |
| **Melanoma** | | | | | | | | |
| UKB, melanoma-specific model  (n = 306,231)^h^ | 1 (ref.) | 0.92 (0.77, 1.11) | 0.83 (0.69, 1.00) | 0.82 (0.65, 1.03) | 0.92 (0.86, 0.99) | 1 (ref.) | 0.97 (0.88, 1.07) | 0.94 (0.69, 1.30) |
| SALT, multivariable model  (n = 34,222)^c^ | 1 (ref.) | 0.69 (0.43, 1.11) | 0.92 (0.58, 1.47) | 0.75 (0.42, 1.34) | 1.00 (0.84, 1.20) | - | - | - |

Abbreviations: SALT, Screening Across the Lifespan Twin Study; UKB, UK Biobank

1. Individuals with missing data on any covariate were excluded.
2. Multivariable model in UKB: adjusted for age (time scale), birth year, sex (except for breast cancer and prostate cancer), baseline assessment center, ethnic background, body mass index, smoking status, alcohol consumption, education level, deprivation index quintiles.
3. Multivariable model in SALT: adjusted for age (time scale), birth year, sex (except for breast cancer and prostate cancerbody mass index, smoking status, alcohol consumption, education level.
4. Breast cancer-specific model in UKB: multivariable model + family history of breast cancer.
5. Prostate cancer-specific model in UKB: multivariable model + family history of prostate cancer.
6. Lung cancer-specific model in UKB: multivariable model + family history of lung cancer.
7. Colorectal cancer-specific model in UKB: multivariable model + family history of colorectal cancer.
8. Melanoma cancer-specific model in UKB: multivariable model + physical activity level, time spent outdoors during summer, use of sun/UV protection, sunburn during childhood, solarium/sunlamp use, ease of skin tanning, skin color, hair color.

* Significant after Bonferroni adjustment at *p*<.005 (i.e., .05/10, considering 2 frailty measures × 5 cancers)

# **Supplementary Figure 1.** Flowchart for the selection of the UK Biobank and SALT analytical samples.

Abbreviations: SALT, Screening Across the Lifespan Twin Study; UKB, UK Biobank

# **Supplementary Figure 2.** Box plot of the frailty index by the frailty phenotype scores in UK Biobank (n = 453,144)


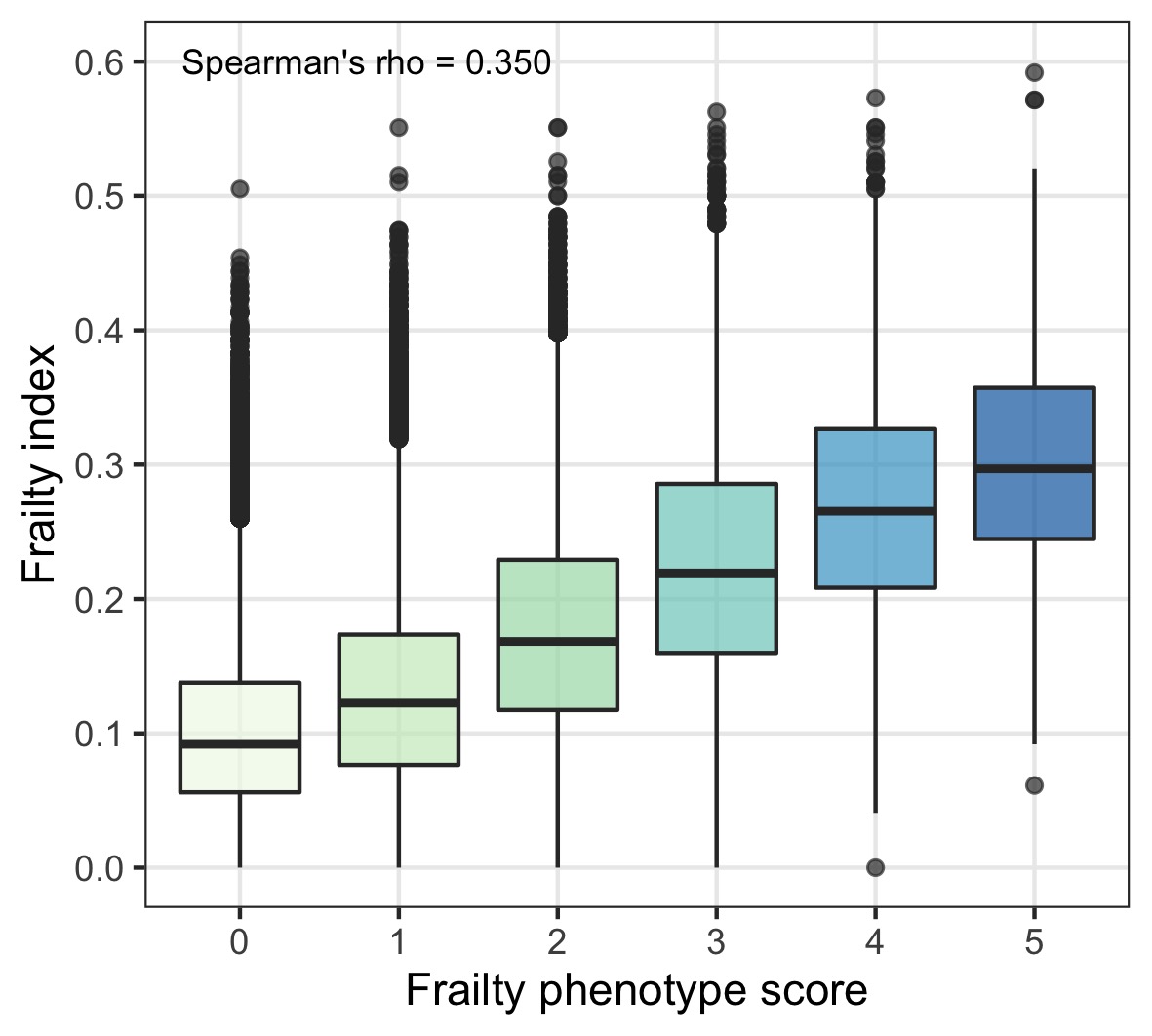


# **Supplementary Figure 3.** Cumulative incidence of cancer over attained age, stratified by frailty status in UK Biobank and SALT


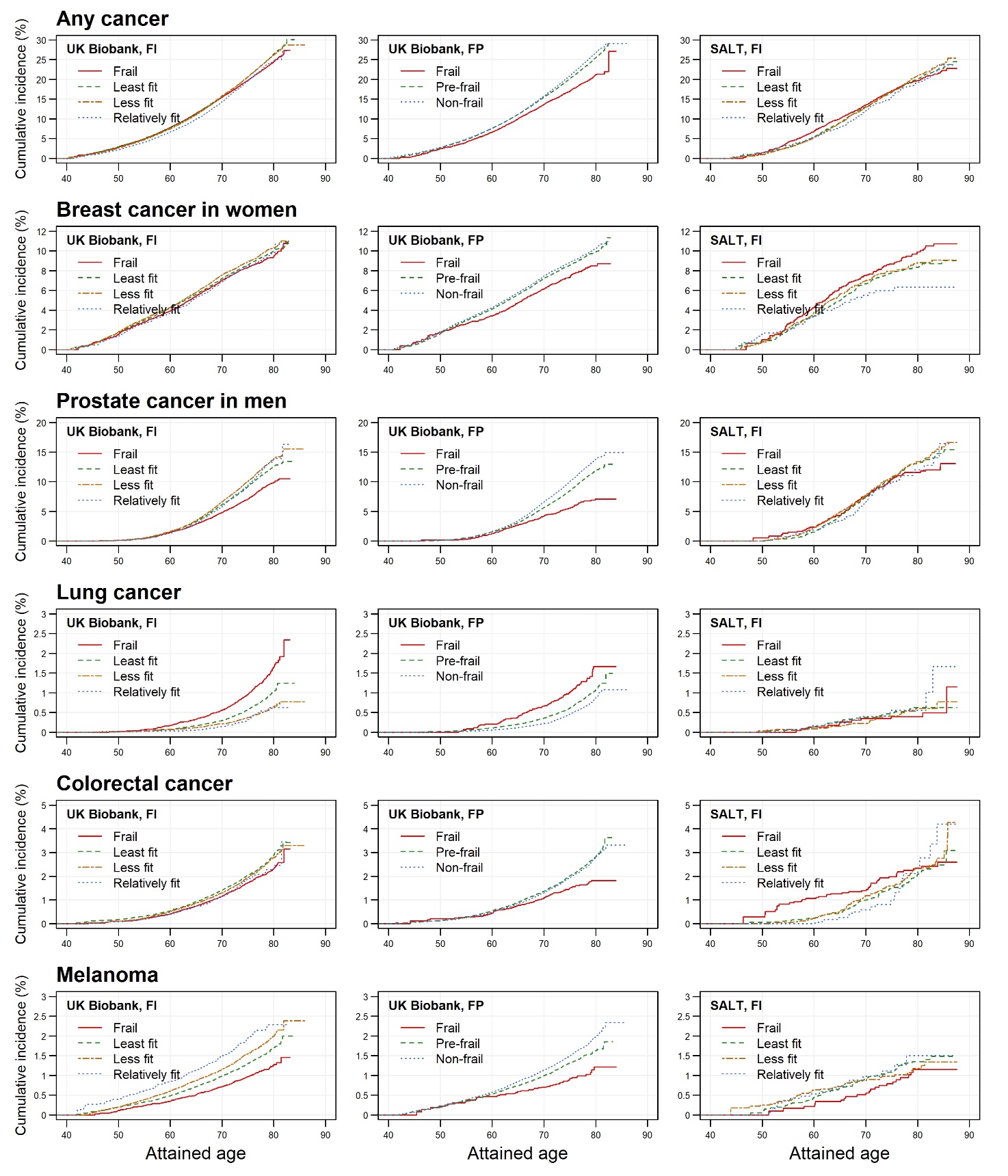


Cumulative incidence was estimated using the Aalen-Johansen estimator, accounting for the competing risk of death. Frailty was assessed using a 49-item FI and a FP in UK Biobank, and by a 44-item FI in SALT.

Abbreviations: FI, frailty index; FP, frailty phenotype; SALT, Screening Across the Lifespan Twin Study.

# **Supplementary Figure 4.** Estimated time-dependent hazard ratios for any cancer by frailty status in UK Biobank and SALT


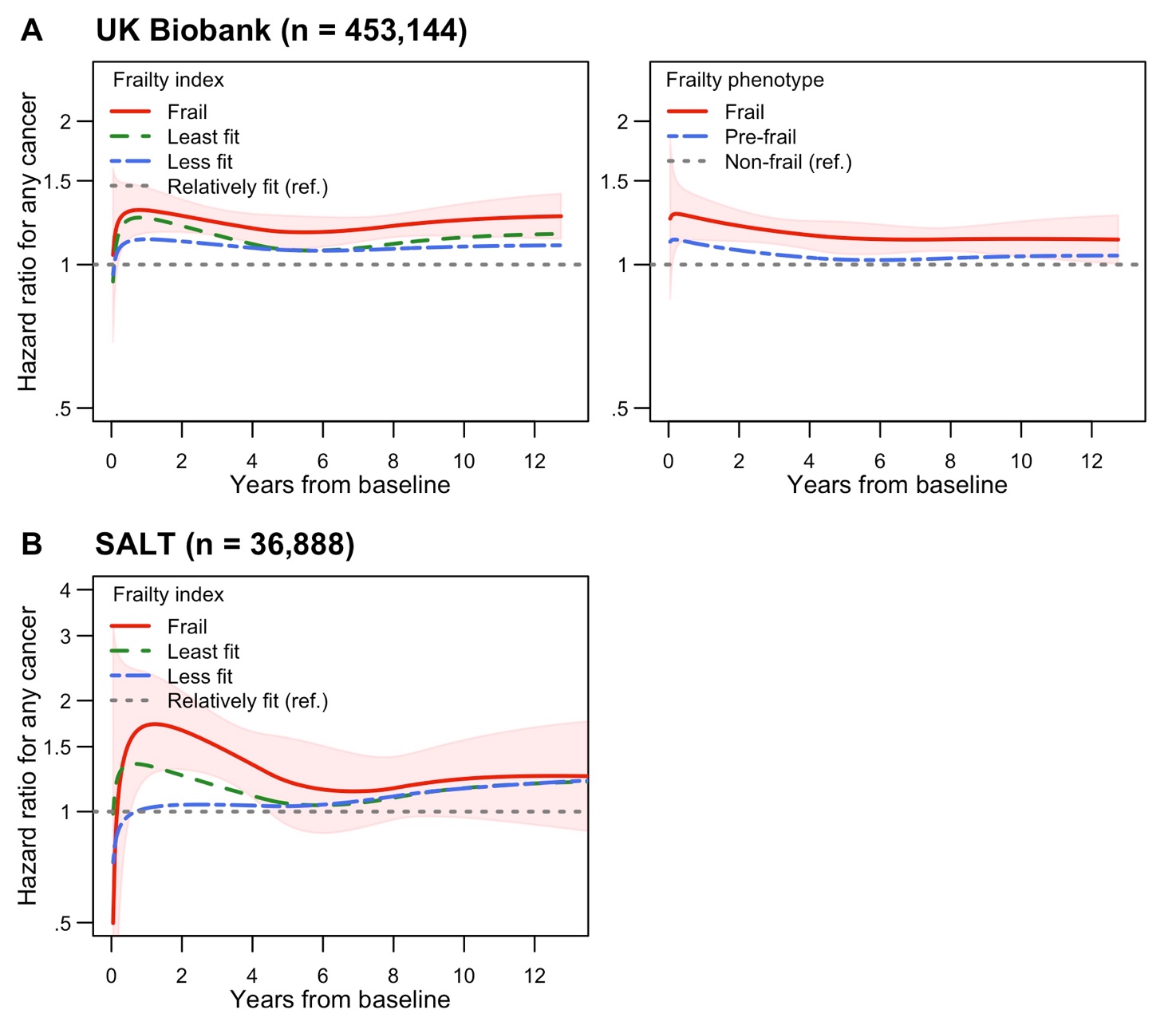


Panel A presents associations between baseline frailty index and frailty phenotype and the risk of any cancer in UK Biobank participants (n = 453,144). Estimates were calculated from flexible parametric survival models using time since measurement as the underlying time scale, where the baseline hazard function was modelled using a 5 degrees-of-freedom natural cubic spline, and the time-dependent effect of frailty using a 3 degrees-of-freedom spline. Models were adjusted for age at baseline, birth year, sex, baseline assessment center, body mass index, ethnic background, smoking status, alcohol consumption, education level, and deprivation index quintiles.

Panel B presents associations between the frailty index and risk of any cancer in SALT participants (n = 36,888); the model was adjusted for age at baseline, birth year, sex, body mass index, smoking status, alcohol consumption, and education level.

The shaded areas indicate 95% confidence intervals for the “frail” group.

Abbreviations: SALT, Screening Across the Lifespan Twin Study.
